# Optimal timing for bilateral total knee arthroplasty: comparing simultaneous and staged procedures at various intervals: a systematic review and network meta-analysis

**DOI:** 10.1530/EOR-2024-0070

**Published:** 2025-01-03

**Authors:** Cheng-Yang Chang, Kun-Han Lee, Jui-Chien Wang, Shang-Wen Tsai, Cheng-Fong Chen, Po-Kuei Wu, Wei-Ming Chen

**Affiliations:** ^1^ Department of Orthopaedics and Traumatology, Taipei Veterans General Hospital, Taipei, Taiwan; ^2^ Department of Orthopaedics, School of Medicine, National Yang Ming Chiao Tung University, Taipei, Taiwan

**Keywords:** bilateral total knee arthroplasty, complication, interval, mortality, outcome, simultaneous, staged

## Abstract

**Purpose:**

- A staged bilateral total knee arthroplasty (BTKA) procedure is considered when a patient is not deemed suitable for simultaneous BTKA due to concerns about the risk of mortality and complications. However, no network meta-analysis has been conducted to compare simultaneous vs staged BTKA procedures with different intervals in terms of postoperative mortality and overall complication rates.

**Methods:**

- Four databases – Medline, Embase, Cochrane Library and Web of Science – were searched from inception to December 19, 2023, for studies comparing patients who underwent staged BTKA with different intervals and simultaneous BTKA. The primary outcome domains were 1-year mortality and 90-day overall complications. Secondary outcomes included neurological, cardiovascular, pulmonary, infectious and venous thromboembolic complications within 90 days.

**Results:**

- Fifteen observational studies were included. Staged BTKA with intervals between 6 weeks and 3 months (odds ratio (OR): 0.69, 95% CI: 0.53–0.91), between 3 and 6 months (OR: 0.67, 95% CI: 0.53–0.84) and longer than 6 months (OR: 0.67, 95% CI: 0.55–0.83) exhibited a lower mortality risk compared to simultaneous BTKA. Staged BTKA with an interval shorter than 6 weeks and longer than 6 months exhibited a higher risk of pulmonary (OR: 1.24, 95% CI: 1.03–1.49; OR: 1.64, 95% CI: 1.10–2.44) and infectious complications (OR: 1.50, 95% CI: 1.15–1.96; OR: 1.52, 95% CI: 1.14–2.02) compared to simultaneous BTKA. An interval between 3 and 6 months ranked best in outcomes of 1-year mortality (*P* score = 0.7849) and 90-day complications (*P* score = 0.7077).

**Conclusions:**

- Staged BKTA with an interval of more than 6 weeks but less than 6 months is associated with a lower risk of postoperative mortality and complications. However, these results should be interpreted with caution due to potential biases inherent in the inclusion of nonrandomized studies.

**Level of Evidence:**

- II

## Introduction

The incidence of bilateral knee arthritis at initial presentation or progression to subsequent contralateral disease can be up to 30–40% (1, 2, 3). Therefore, the choice between simultaneous and staged bilateral total knee arthroplasty (BTKA) is a common clinical scenario. The considerations for choosing either simultaneous or staged BTKA were mainly based on costs, rates of complication, readmission and mortality (4, 5, 6, 7, 8, 9, 10, 11). Simultaneous BTKA was associated with a shorter overall length of stay, lower total medical costs and reimbursement from the health insurance system compared to staged BTKA (4, 6, 7). However, simultaneous BTKA was associated with an increased risk of 90-day mortality rate, deep vein thrombosis, pulmonary embolism and neurologic complications compared to staged BTKA, according to the results from recent meta-analyses (8, 9). In terms of the rates of complications and mortality, staged BTKA seems to be a safer option than simultaneous BTKA.

Among the studies that compared complication and mortality rates between staged and simultaneous BTKA, or different intervals between staged BTKA, the definition of these intervals was generally within 12 months, but it varied across studies (12, 13, 14, 15, 16, 17, 18, 19, 20, 21, 22, 23, 24, 25). The impact of different intervals on these outcomes remains controversial (12, 15, 17, 18, 20, 22, 23, 24). Some studies have validated that short intervals (within or up to 1 week and, 1–6 weeks) between staged BTKA were associated with a higher risk of having any or major complications (17, 22, 23), while others found no difference in risk among different intervals (less than 1 week, 1–3, 3–6, 6–9 and 9–12 months) (15, 18, 21, 24). To conclude that staged BTKA might be a safer alternative to simultaneous BTKA in terms of a lower risk of complications and mortality, it is crucial to differentiate the risks among different intervals between staged BTKA and simultaneous BTKA as they were largely heterogeneous (12, 13, 14, 15, 16, 17, 18, 19, 20, 21, 22, 23, 24). We conducted this meta-analysis because of the lack of comprehensive network meta-analyses investigating the impact of the interval between staged BTKA on outcomes.

Our primary objective was to validate the optimal timing for BTKA concerning lower rates of mortality within 1 year and overall complications within 90 days. The secondary objective was to compare the risk of each common complications (including cardiovascular, pulmonary, neurologic, infection and venous thromboembolic events) across different intervals in staged BTKA and simultaneous BTKA.

## Materials and methods

### Data sources and searches

This systematic review adhered to the PRISMA 2020 Extension guideline (Supplementary Table S1 (see the section on Supplementary materials given at the end of the article)). The review protocol was registered in PROSPERO, the international prospective register of systematic reviews, under Registration Number CRD42024541190. Two independent reviewers (CYC and KHL) conducted a comprehensive search across four databases: Medline, Embase, Cochrane Library and Web of Science, from inception to December 19, 2023. Disagreements regarding study inclusion were resolved by a third author (SWT), who made the final decisions. Search terms included ‘bilateral total knee arthroplasty’ OR ‘bilateral TKA’ OR ‘bilateral total knee replacement’ OR ‘bilateral TKR’ AND ‘simultaneous’ OR ‘one-staged’ OR ‘staggered’ OR ‘staged’ OR ‘staging’ OR ‘non-simultaneous’ OR ‘two-staged’. The reference lists of selected studies were screened, and the search was not limited by study size or language.

### Study selection

The PICO criteria (Supplementary Table S2) for the study population are defined as follows: P (patient) includes patients undergoing BTKA. I (intervention) refers to the different time intervals between staged BTKA procedures. C (comparison) involves the simultaneous BTKA procedure. O (outcome) includes complications within 90 days and mortality within 1 year after the procedure.

Two reviewers (CYC and KHL) assessed studies for eligibility based on specific criteria: i) studies involving human subjects of any age and gender; ii) studies on populations that underwent either simultaneous (both procedures under one anesthesia) or staged (procedures under separate anesthesia) BTKA, comparing clinical outcomes or postoperative complications; iii) studies comparing different time intervals between BTKA surgeries, time intervals were defined as simultaneous, shorter than 6 weeks, between 6 weeks and 3 months, between 3 and 6 months and longer than 6 months, with at least two comparison groups in the study; iv) study designs encompassing randomized control trials (RCTs) or observational cohort studies (either prospective or retrospective); and v) studies reporting on postoperative complications within 90 days and mortality within 1 year.

The primary outcomes included mortality and overall complications. Overall complications were defined as the total number of complications associated with each individual surgery within 90 days, as reported in each study, excluding mortality. These complications encompassed surgical, perioperative, wound-related issues and medical complications, including neurologic, cardiovascular, pulmonary, infectious, venous thromboembolic, renal, cerebrovascular and gastrointestinal events. The secondary outcomes focused on common complications, such as neurologic, cardiovascular, pulmonary, infectious and venous thromboembolic events, within 90 days. Neurologic complications included cerebrovascular disease, transient ischemic attack, confusion and delirium. Cardiovascular complications included congestive heart failure, arrhythmia and acute coronary events. Pulmonary complications involved the need for mechanical ventilation, pulmonary compromise, respiratory failure, pulmonary embolism and pneumonia. Infectious events comprised wound infection, deep infection, nosocomial infection, urinary tract infection, pneumonia, sepsis and septic shock. Venous thromboembolism included deep vein thrombosis and pulmonary embolism.

If multiple studies using the same database were identified, we selected the study with more comprehensive data or the one that included a larger number of patients. Authors of potentially relevant studies were contacted for additional information. Exclusions were made for nonhuman studies, systematic reviews, meta-analyses, case series, case reports, editorial comments and studies comparing unilateral to BTKA.

### Data extraction and quality assessment

Two investigators (CYC and KHL) extracted data using a standardized form that included journal title, first author, year of publication, data source, study design, sample size, treatment arms, follow-up duration and patient characteristics. The quality of the studies was evaluated using the Cochrane Risk of Bias Tool 2.0 (ROB 2.0; https://sites.google.com/site/riskofbiastool/welcome/rob-2-0-tool) for randomized controlled trials (26) and the Newcastle–Ottawa Scale (NOS) for nonrandomized cohort studies (27). According to the ROB 2.0 framework (26), a study is considered low risk if all domains are rated as low risk. It is classified as having some concerns if one domain raises issues and as high risk if any domain is judged as high risk or if there are multiple concerns that significantly reduce confidence in the results. For nonrandomized cohort studies, those scoring 5–6 on the NOS were classified as moderate quality, while those scoring 0–4 were considered low quality. This criterion was guided by comparable literature and achieved through consensus among our research team (28, 29). Those classified as high risk of bias or low-quality were excluded. Discrepancies during the reference search, study identification and data extraction were initially resolved through discussion between the two reviewers. Any unresolved issues were brought to a third investigator (SWT), who made the final decisions.

### Statistical analysis

We extracted the reported patient number of each complication from articles or calculated them using the incidence rate and total number of patient if the patient number was not available for analysis. They were then transformed into odds ratios (ORs) with 95% confident intervals (95% CIs). Statistical significance was set at *P* value < 0.05 in the two-tailed test. For network meta-analysis, the R package v4.3.2 (https://cran.r-project.org/src/base/R-4/) with the ‘netmeta’ package and ‘CINeMA’ website was used to identify the optimal treatment with result of primary outcome (30). Network graph was drawn to explore the geometry of the network. A random-effects model was utilized; it was based on a graph theoretical approach using the frequentist statistical framework. The consistency was calculated for between-design heterogeneity under the assumption of a full design-by-treatment.

The Egger’s test was implemented to evaluate the potential publication bias of the included studies. The consistency was calculated to assess the between-design heterogeneity. The *P* score of the treatment ranking, which was demonstrated to be equivalent to the SUCRA score, was applied to incorporate the ranking and possibility of a certain treatment being the best (31).

## Results

### Literature search

**Figure 1:**
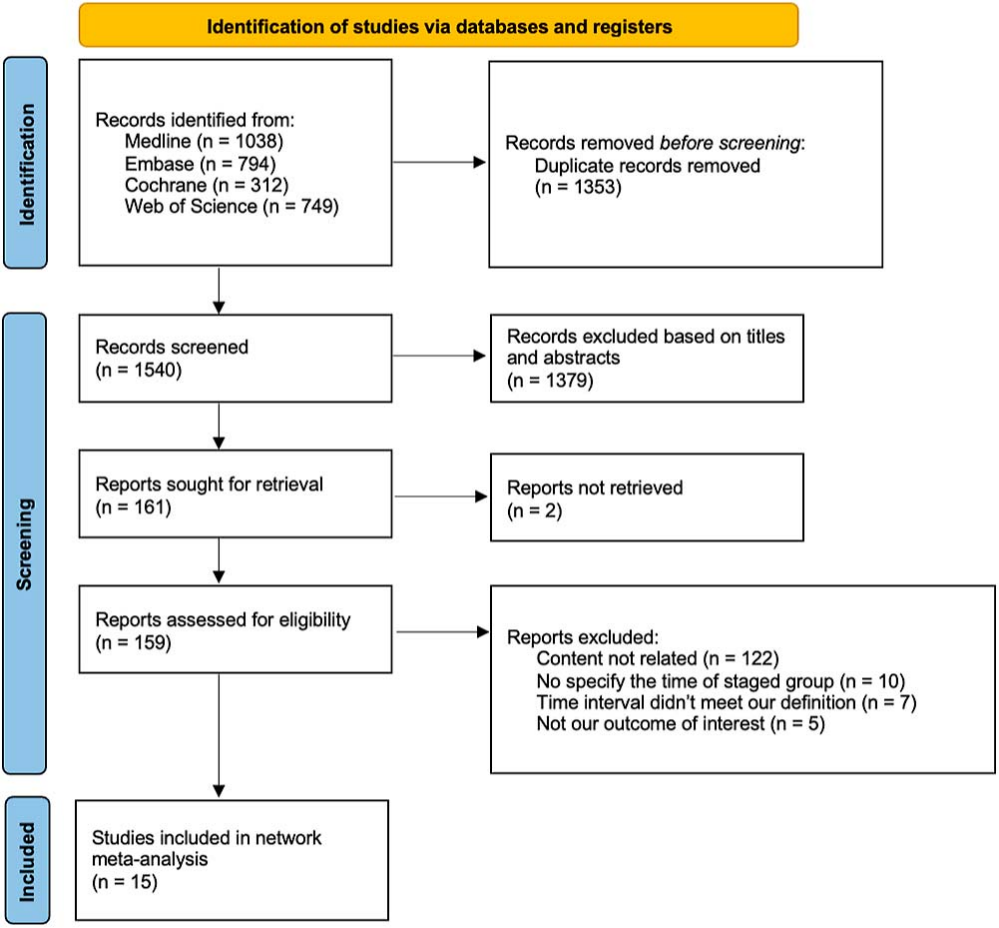
PRISMA 2020 flow diagram.

**Figure 2:**
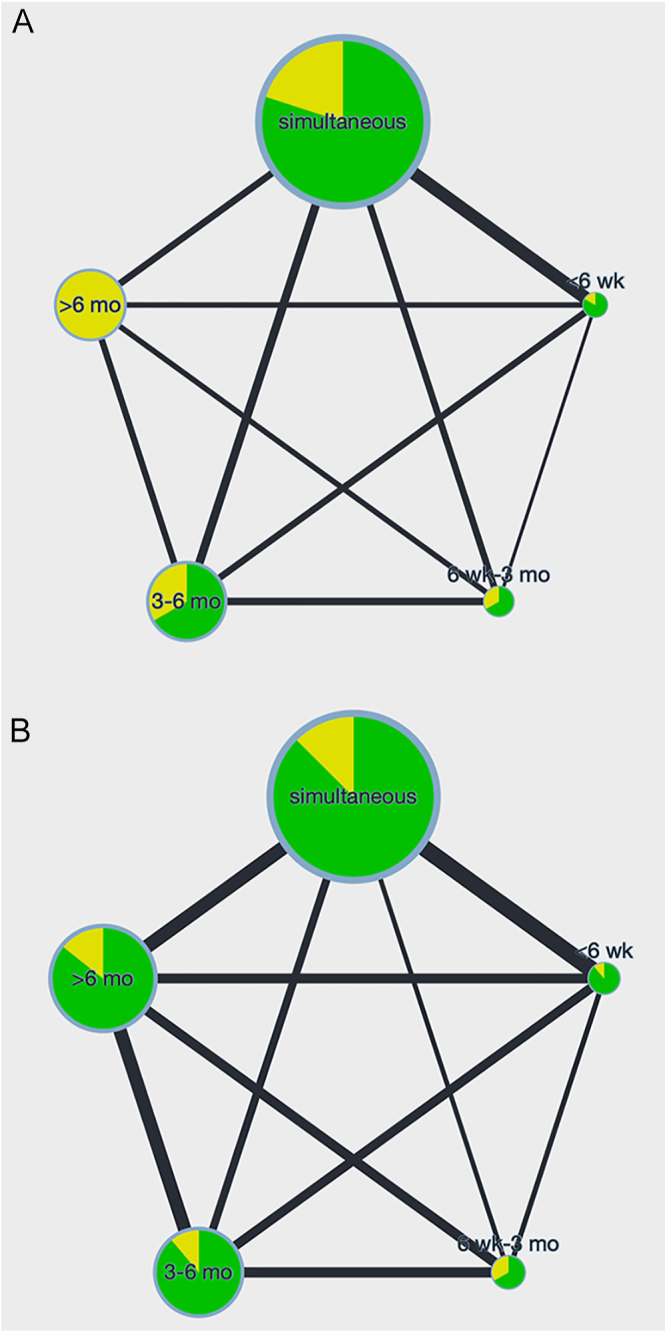
Network plot of primary complications. (A) Mortality and (B) overall complications. The plot illustrates comparisons of different time intervals between BTKA in a network diagram. The size of each node reflects the sample size of studies for that interval. The colors within the nodes indicate the assessed risk of bias for each group. The thickness of the lines represents the sample sizes involved in the comparisons between the two time intervals.

Within the 1,540 identified records, 161 papers were retrieved for full-text review after screening abstracts and titles. The authors of some possibly eligible studies were contacted, one of which replied with more detailed information (4). Of these, no RCTs met our inclusion criteria, and 15 observational cohort studies were included in the final analysis (Fig. 1). The detailed search strategy and keywords used were listed in Supplementary Table S3. The baseline information of the 15 observational studies is demonstrated in Table 1 (4, 12, 13, 14, 15, 16, 17, 18, 19, 20, 21, 22, 23, 24, 25). Of note, these studies were published between 1997 and 2023. Regarding the featured characteristics (Supplementary Table S4), the mean age varied from 63.0 to 73.0 years. Network graphs were provided to enable visualization of the geometry of the treatment network (Fig. 2A and B and Supplementary Fig. S1A, B, C, D, and E). According to the network graphs, both mortality and overall complications presented direct comparison between each time interval, while mortality showed less sample size included and higher risk of bias. The NOS was employed to assess the quality of 15 observational cohort studies. Fourteen of these studies achieved scores ranging from 7–9, denoting high quality, while one study scored a 6, indicating moderate quality based on predefined criteria. No studies were excluded due to low quality. The details of the bias assessments are available in Table S5.

**Table 1:** Characteristics of included studies.

Study	Country	Data Source	Study design	Time intervals	Arms	Outcome
Ritter *et al.* (12)	USA	Health Care Financing Administration data	RCS	Simultaneous; <6 weeks; 6 weeks to 3 months; 3–6 months; 6–12 months	Simultaneous; <6 weeks; 6 weeks to 3 months; 3–6 months; >6 months	1, 2, 4, 6
Forster *et al.* (13)	Australia	N/A	RCS	Simultaneous; 7 days; 5–68 months	Simultaneous; <6 weeks	2, 3, 7
Courtney *et al.* (14)	USA	Hospital of the University of Pennsylvania	RCS	Simultaneous; 1 week	Simultaneous; <6 weeks	1, 2, 3, 4, 5, 6, 7
Liu *et al.* (17)	USA	Nationwide Inpatient Sample data	RCS	Same day; 1–3 days; 4–7 days	Simultaneous; <6 weeks	1, 2, 3, 4, 5, 6, 7
Koh *et al.* (16)	S Korea	Seoul St. Mary’s Hospital	RCS	Simultaneous; 1 week	Simultaneous; <6 weeks	2, 3, 4, 5, 6, 7
Chen *et al.* (15)	USA	Thomas Jefferson University Hospital	RCS	21–90 days; 91–180 days; 181–270 days; 271–360 days	3–6 months; >6 months	2, 4, 5, 6, 7
Yeh *et al.* (18)	Singapore	Singapore General Hospital	RCS	31–90 days; 9–180 days; 181–270 days; 271–365 days	3–6 months; >6 months	2, 4, 5, 6, 7
Chua *et al.* (25)	Australia	AOANJRR	RCS	Simultaneous; 1 day to 6 weeks; 6 weeks to 3 months; 3–6 months	Simultaneous; <6 weeks; 6 weeks to 3 months; 3–6 months	1
Richardson *et al.* (19)	USA	PearlDiver Patient Records Database	RCS	Simultaneous; <3 months; 3–6 months; 6–12 months	Simultaneous; 3–6 months; >6 months	2, 4, 5, 6, 7
Crawford *et al.* (20)	USA	The Ohio State University Wexner Medical Center	RCS	3–6 weeks; 7–12 weeks; 13–24 weeks; >24 weeks	<6 weeks; 6 weeks to 3 months; 3–6 months; >6 months	2, 7
Mardani-Kivi *et al.* (21)	Iran	N/A	RCS	Simultaneous; 3 days; 3–12 months	Simultaneous; <6 weeks	1, 2, 3, 6, 7
Xu *et al.* (22)	China	The Affiliated Hospital of Qingdao University	PCS	7 days; 90–120 days	<6 weeks; 3–6 months	2, 3, 4, 5
Sun *et al.* (24)	China	West China Hospital	RCS	2–6 months; 6–12 months; >12 months	3–6 months; >6 months	2, 4, 6, 7
Agarwal *et al.* (23)	USA	PearlDiver Patient Records Database	RCS	1–6 weeks; 7–12 weeks; 13–18 weeks; 19–24 weeks	<6 weeks; 6 weeks–3 months; 3–6 months	1, 2, 3, 4, 5, 6, 7
Chou *et al.* (4)	Taiwan	Taipei Veterans General Hospital	RCS	Simultaneous; 3–6 months; >6 months	Simultaneous; 3–6 months; >6 months	2, 3, 4, 5, 6, 7

1 = mortality; 2 = all complications; 3 = neurologic complications; 4 = cardiovascular complications; 5 = pulmonary complications; 6 = infection; 7 = venous thromboembolism; AOANJRR, Australian Orthopaedic Association National Joint Replacement Registry; N/A = not applicable; RCS, retrospective cohort study; PCS, prospective cohort study.

### Network meta-analysis among different time intervals and simultaneous BTKA

**Figure 3:**
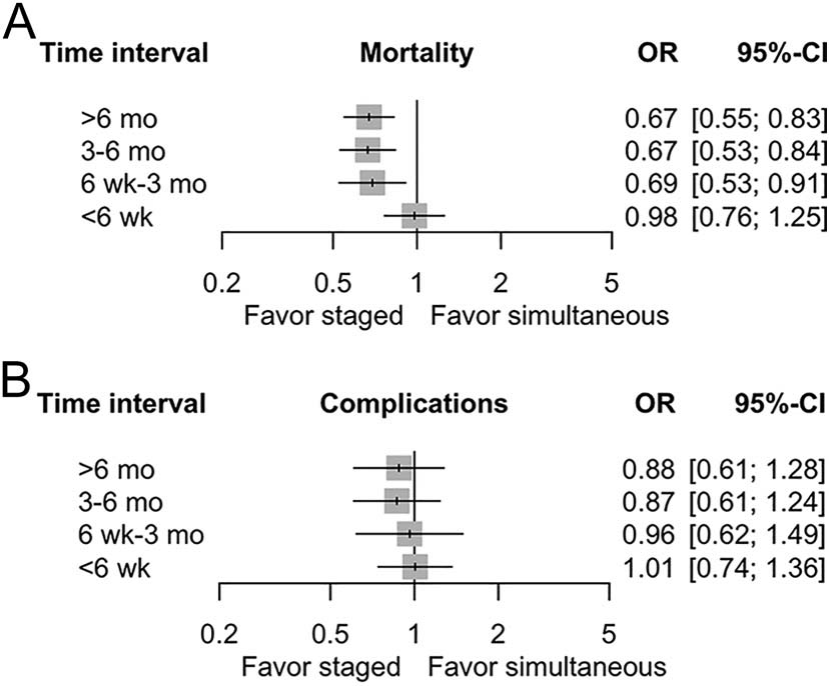
Forest plot for different outcomes. (A) Mortality and (B) overall complications. OR, odds ratio; CI, confidence interval.

A total of 15 observational studies were analyzed in the network meta-analysis and were presented using forest plots and league tables. The results from the forest plot (Fig. 3A) indicate that BTKA with intervals longer than 6 months (OR: 0.67, 95% CI: 0.55–0.83), between 3 and 6 months (OR: 0.67, 95% CI: 0.53–0.84) and between 6 weeks and 3 months (OR: 0.69, 95% CI: 0.53–0.91) are associated with reduced mortality risk compared to simultaneous BTKA. However, intervals shorter than 6 weeks did not show a significant difference in mortality risk when compared to simultaneous BTKA. According to the league tables, time intervals longer than 6 months (OR: 0.69, 95% CI: 0.54–0.88), between 3 and 6 months (OR: 0.68, 95% CI: 0.52–0.89) and between 6 weeks and 3 months (OR: 0.71, 95% CI: 0.52–0.96) also showed lower risk in mortality with significant difference when compared to the group with intervals of less than 6 weeks (Table 2). Regarding overall complications, the risk associated with staged BTKA at any interval compared to simultaneous BTKA showed no significant difference (Fig. 3B). This finding is also supported by the league table, displaying no statistically significant differences in the risk of overall complications between any of the arms, in both direct and combined comparisons (Table 2).

**Table 2:** League table comparing outcomes between staged BTKA with different time intervals and simultaneous BTKA. The league table presents odds ratios (ORs) with 95% CI for comparing different time intervals between bilateral knee arthroplasties. The upper half of the triangle shows ORs for direct comparisons available within our network. The lower half of the triangle includes ORs for estimated effect sizes based on both direct and indirect comparisons. In the upper grids, each box compares the time interval defined by the row with that defined by the column. Otherwise, in the lower grids, each box compares the time interval defined by the column with that defined by the row. The bold text highlights the five study arms in this research.

A: Mortality				
**>6 months**	0.98 (0.78; 1.24)	0.95 (0.73; 1.25)	0.69 (0.53; 0.88)*	0.69 (0.56; 0.86)*
1.01 (0.81; 1.27)	**3–6 months**	0.98 (0.73; 1.32)	0.69 (0.52; 0.90)*	0.68 (0.54; 0.85)*
0.97 (0.74; 1.28)	0.96 (0.72; 1.29)	**6 weeks to 3 months**	0.71 (0.52; 0.96)*	0.71 (0.54; 0.94)*
0.69 (0.54; 0.88)*	0.68 (0.52; 0.89)*	0.71 (0.52; 0.96)*	**<6 weeks**	0.98 (0.77; 1.26)
0.67 (0.55; 0.83)*	0.67 (0.53; 0.84)*	0.69 (0.53; 0.91)*	0.98 (0.76; 1.25)	**Simultaneous**
B: Overall complications				
**>6 months**	0.90 (0.64; 1.25)	1.28 (0.74; 2.22)	1.25 (0.72; 2.15)	0.92 (0.59; 1.46)
1.02 (0.74; 1.40)	**3–6 months**	0.81 (0.52; 1.25)	0.74 (0.50; 1.11)	0.98 (0.61; 1.57)
0.92 (0.59; 1.42)	0.90 (0.60; 1.35)	**6 weeks to 3 months**	0.95 (0.61; 1.46)	0.99 (0.48; 2.07)
0.87 (0.60; 1.27)	0.86 (0.61; 1.21)	0.96 (0.64; 1.44)	**<6 weeks**	0.98 (0.70; 1.38)
0.88 (0.61; 1.28)	0.87 (0.61; 1.24)	0.96 (0.62; 1.49)	1.01 (0.74; 1.36)	**Simultaneous**

* denotes statistically significant difference.

Major complications, including neurologic, cardiovascular, pulmonary, infectious and venous thromboembolic events, were individually evaluated as secondary outcomes (Supplementary Fig. S2A, B, C, D, and E). In terms of pulmonary complications, staged BTKA with intervals shorter than 6 weeks (OR: 1.24, 95% CI: 1.03–1.49) and longer than 6 months (OR: 1.64, 95% CI: 1.10–2.44) exhibited a higher risk compared to simultaneous BTKA (Supplementary Fig. S2C). The league table presents consistent findings across both combined and direct comparisons (Table S6C). Furthermore, the league table (Table S6C) indicates that in combined comparisons, a time interval longer than 6 months is associated with a higher risk of pulmonary complications compared to a time interval of 3–6 months. Regarding the risk of infection, staged BTKA with intervals shorter than 6 weeks (OR: 1.50, 95% CI: 1.15–1.96) and longer than 6 months (OR: 1.52, 95% CI: 1.14–2.02) showed a higher risk compared to simultaneous BTKA (Supplementary Fig. S2D). The league table also presents similar findings in both combined and direct comparisons (Table S6D). The risk of neurologic, cardiovascular and venous thromboembolic events was not different between staged BTKA with any interval and simultaneous BTKA.

#### Publication bias, consistency and quality of evidence

The assessment of publication bias using Egger’s test (Supplementary Fig. S3A and B) and consistency using the Q statistic under a full design-by-treatment interaction random-effects mode (Supplementary Table S7) showed that no statistically significant publication bias was detected with Egger’s test (*P* = 0.828) in studies reporting complications. Similarly, no statistically significant inconsistency was found in mortality (*P* = 0.8564) and overall complications (*P* = 0.3835). However, a statistically significant publication bias was observed (*P* = 0.0344) in studies reporting risk of mortality. The reporting bias in studies discussing mortality may be attributed to a relatively small number of studies (*n* = 6). The results of the quality-of-evidence assessments conducted using CINeMA are presented in Supplementary Table S8A and B, showing a range of evidence quality from high to low in studies comparing different time intervals for the occurrence of overall complications and evidence of quality from moderate to low in studies discussing mortality.

#### *P* score ranking

Ranking with the *P* score in the random-effects model, using the results of network meta-analysis with 15 observational studies, illustrated the potential optimal treatment options for different outcomes. In terms of mortality, staged BTKA with intervals longer than 6 weeks ranked higher, including between 3 and 6 months (*P* score: 0.7849), longer than 6 months (*P* score: 0.7592) and between 6 weeks and 3 months (*P* score: 0.7004). Regarding overall complications, staged BTKA with intervals longer than 6 weeks consistently ranked higher than an interval shorter than 6 weeks (*P* score: 0.3329) and simultaneous BTKA (*P* score: 0.3528). The *P* score for staged BTKA with intervals between 3 and 6 months, longer than 6 months and between 6 weeks and 3 months were 0.7077, 0.6548, and 0.4518, respectively (Supplementary Table S9).

## Discussion

This work represents the first network meta-analysis, to our knowledge, to investigate the optimal interval between staged BTKA in terms of lower risks of complication and mortality. The main findings were as follows: i) the risk of mortality was lower for intervals longer than 6 weeks (including 6 weeks to 3 months, 3–6 months and >6 months) compared to intervals less than 6 weeks and simultaneous BTKA; ii) the risk of overall complications was not different between staged BTKA with any interval and simultaneous BTKA; and iii) intervals of less than 6 weeks and more than 6 months were associated with a higher risk of infection and pulmonary complications, but showed no difference in the risk of cardiovascular, neurologic complications and venous thromboembolism.

According to the statement from the Consensus Conference on Bilateral Total Knee Arthroplasty Group in 2013, 81% of participants agreed on an interval of at least 3 months for a second TKA if a patient is not deemed a candidate for simultaneous BTKA. However, there is a paucity of data at the time of this statement, and no definitive conclusions can be drawn (32). Ritter and coworkers conducted a database study and compared the cumulative mortality rates for up to 2 years among patients undergoing simultaneous and staged BTKA with different intervals (<6 weeks, 6 weeks to 3 months, 3–6 months and 6–12 months). They found that simultaneous BTKA was associated with a higher mortality rate (0.99–1.93%) in the early postoperative period (approximately 3–6 months), compared to each interval of stage BTKA (0.29–1.75%). Notably, there were no significant differences in mortality rates observed in postoperative 1 year and beyond (12). In another database study, Liu and coworkers compared the perioperative mortality rates among patients who underwent simultaneous BTKA and staged BTKA with short intervals (1–3 and 4–7 days). The overall rates were very low (0.07–0.23%) and not different between these groups (17). Chua and coworkers analyzed data from the Australian Orthopaedic Association National Joint Replacement Registry (AOANJRR) and compared the 30-day mortality rate among patients undergoing simultaneous BTKA and staged BTKA with different intervals (<6 weeks, 6 weeks to 3 months and 3–6 months). The 30-day mortality rates in the simultaneous and staged BTKA group were 0.17 and 0.06%, respectively. When adjusted for age and gender, there was a lower risk of 30-day mortality (adjusted OR (aOR): 0.30, 95% CI: 0.13–0.72) in the staged 6-week to 3-month group. The risk was not different between simultaneous BTKA and other intervals (25). Despite the low overall mortality rate following simultaneous or staged BTKA, the risk of mortality is higher in the early postoperative period (12, 17, 25). This meta-analysis revealed that staged BTKA with intervals longer than 6 weeks was associated with a lower risk of mortality compared to staged BTKA with an interval less than 6 weeks and simultaneous BTKA, which differs from the statement provided by the Consensus Conference (32). Simultaneous BTKA may impose greater physiological stress on patients, potentially increasing the risk of mortality (33). Some studies suggest that this increased risk could be linked to a higher incidence of pulmonary embolism (9, 34). Findings from this meta-analysis indicate that staging BTKA procedures with an interval of more than 6 weeks could help mitigate this risk. However, it is important to note a potential bias in these results due to the retrospective design of the included studies. Specifically, patients who died after the first TKA or experienced major complications were less likely to proceed with the second TKA, which may skew outcomes in favor of staged procedures (8, 28).

Another outcome domain to be addressed is the major complications following simultaneous or staged BTKA with different intervals, including cardiovascular events, neurologic events, pulmonary complications, infection and venous thromboembolism (12, 17, 19, 20, 23). Agarwal and coworkers conducted a database study and compared the risk of overall and major complications among patients who underwent staged BTKA with different intervals (1–6, 7–12, 13–18 and 19–24 weeks). When compared to staged 1–6-week group, the staged 7–12-week group showed a borderline lower risk of all and major complications. The risks were significantly lower in staged BTKA with intervals of 13–18 and 19–24 weeks. Notably, there was a decreasing trend of OR for all (7–12 weeks: 0.91, 13–18 weeks: 0.41 and 19–24 weeks: 0.33) and major complications (7–12 weeks: 0.82, 13–18 weeks: 0.67 and 19–24 weeks: 0.62) as the intervals lengthened, when compared to the staged 1–6-week group (23). In the database study conducted by Richardson *et al.*, 7,747 patients who underwent simultaneous or staged BTKA with intervals within 3 months, 3–6 months and 6–12 months were included for analysis. Compared to the simultaneous BTKA group, the risk of infection was higher in all staged groups, with an adjusted OR ranging from 1.89 to 3.19. The authors hypothesized that additional exposure to infectious organisms associated with two separate procedures and increased total hospitalization might explain this finding. However, the study did not compare infection risks between intervals. In addition, the risk of venous thromboembolism, sepsis and myocardial infarction was not different among the groups (19). In contrast to the findings of Richardson *et al.*, Ritter *et al.* demonstrated that the risk of infection was lower in the staged BTKA group with an interval of 6 weeks to 3 months, compared to the simultaneous BTKA group (12). Liu *et al.* validated that in a staged BTKA group with a short interval (1–3 days), there was a higher risk of any major complications (aOR: 1.38, 95% CI: 1.14–1.67), cardiac complication (aOR: 1.32, 95% CI: 1.06–1.65), pulmonary compromise (aOR: 2.07, 95% CI: 1.45–2.96) and pneumonia (aOR: 1.68, 95% CI: 1.02–2.79), compared to simultaneous BTKA group. Interestingly, the risk of any major complication did not differ between the other staged BTKA groups with an interval of 4–7 days and the simultaneous BTKA group (17). Other studies have not shown a difference in the risk of overall or any complications between simultaneous and staged BTKA with different intervals (14, 15, 16, 18, 24). In this meta-analysis, intervals shorter than 6 weeks or longer than 6 months were associated with a higher risk of infection and pulmonary complications. However, there were no significant differences in overall complications, cardiovascular, neurologic complications or venous thromboembolism among the groups. Given that most of the included studies were retrospective, we hypothesized that patients who experienced medical complications after the first TKA might have influenced the timing of the second procedure, potentially resulting in longer intervals. As reported by Crawford *et al*., staged BTKA with an interval longer than 24 weeks showed the highest rate of medical complications following the first knee surgery (20). This meta-analysis suggests that intervals of less than 6 weeks or longer than 6 months are not recommended due to an increased risk of infection and pulmonary complications. However, these findings should be interpreted with caution due to potential biases in the allocation of patients to staged BTKA groups.

There were several limitations of this meta-analysis. First, most of the included studies were retrospective cohort studies. The allocation to simultaneous or staged BTKA with specific intervals was not randomly or prospectively designated. Specifically, the intervals between staged BTKA could have been affected by factors such as experiencing medical or surgical complications following the first knee procedure or suboptimal outcomes of the first procedure that the patient refused or wished to postpone the second procedure (20). Second, the data on some other important outcome domains within this topic, such as surgical complications, implant survival and functional outcomes, were inadequate for quantitative analysis or drawing conclusion (13, 18, 19, 20, 21, 22, 23). Third, at the study level, a risk of bias was identified in some studies, potentially affecting the credibility of our findings. Fourth, we only included studies reporting 90-day complications and 1-year mortality. Long-term complications and mortality rates between simultaneous and staged BTKA with different intervals remain undetermined.

## Conclusion

This systematic review and network meta-analysis found that staged BTKA with an interval longer than 6 weeks was associated with a lower risk of mortality compared to the staged BTKA with an interval shorter than 6 weeks and simultaneous BTKA. In addition, the patients who underwent staged BTKA with an interval shorter than 6 weeks and longer than 6 months had a higher risk of pulmonary complications and infection, indicating that an interval longer than 6 weeks but less than 6 months might be favorable for the staged BTKA procedure. The results should be interpreted with caution due to the inherent risk of bias associated with the design of the included nonrandomized cohort studies.

## Supplementary materials



## ICMJE statement of interest

The authors declare that there is no conflict of interest that could be perceived as prejudicing the impartiality of the work reported.

## Funding statement

This work did not receive any specific grant from any funding agency in the public, commercial or not-for-profit sector.

## Author contribution statement

All authors made substantial contributions to the conception and design, acquisition of data, analysis, interpretation, drafting this work and critically revising it for important intellectual content.

## References

[1] McMahon M & Block JA. The risk of contralateral total knee arthroplasty after knee replacement for osteoarthritis. J Rheumatol 2003 30 1822–1824. (https://www.jrheum.org/content/30/8/1822) 12913941

[2] Santana DC, Anis HK, Mont MA, et al. What is the likelihood of subsequent arthroplasties after primary TKA or THA? Data from the osteoarthritis initiative. Clin Orthop Relat Res 2020 478 34–41. (10.1097/corr.0000000000000925) 31425280 PMC7000041

[3] Cheng YC, Wu PK, Chen CF, et al. Analysis of learning curve of minimally invasive total knee arthroplasty: a single surgeon’s experience with 4017 cases over a 9-year period. J Chin Med Assoc 2019 82 576–583. (10.1097/jcma.0000000000000118) 31021883 PMC13048205

[4] Chou TFA, Ma HH, Tsai CW, et al. The safety and cost-analysis of simultaneous versus staged bilateral total knee arthroplasty in a Taiwan population. J Chin Med Assoc 2023 86 494–498. (10.1097/jcma.0000000000000892) 36740745 PMC12755661

[5] Lee KH, Chang WL, Tsai SW, et al. The impact of charlson comorbidity index on surgical complications and reoperations following simultaneous bilateral total knee arthroplasty. Sci Rep 2023 13 6155. (10.1038/s41598-023-33196-x) 37061607 PMC10105729

[6] Lin AC, Chao E, Yang CM, et al. Costs of staged versus simultaneous bilateral total knee arthroplasty: a population-based study of the Taiwanese National Health Insurance Database. J Orthop Surg Res 2014 9 59. (10.1186/s13018-014-0059-6) 25023777 PMC4223718

[7] Goh GS, Sutton RM, D’Amore T, et al. A time-driven activity-based costing analysis of simultaneous versus staged bilateral total hip arthroplasty and total knee arthroplasty. J Arthroplasty 2022 37 S742–S747. (10.1016/j.arth.2022.01.048) 35093545

[8] Makaram NS, Roberts SB & Macpherson GJ. Simultaneous bilateral total knee arthroplasty is associated with shorter length of stay but increased mortality compared with staged bilateral total knee arthroplasty: a systematic review and meta-analysis. J Arthroplasty 2021 36 2227–2238. (10.1016/j.arth.2021.01.045) 33589276

[9] Liu L, Liu H, Zhang H, et al. Bilateral total knee arthroplasty: simultaneous or staged? A systematic review and meta-analysis. Medicine 2019 98 e15931. (10.1097/md.0000000000015931) 31145362 PMC6708906

[10] Pai FY, Chang WL, Tsai SW, et al. Pharmacological thromboprophylaxis as a risk factor for early periprosthetic joint infection following primary total joint arthroplasty. Sci Rep 2022 12 10579. (10.1038/s41598-022-14749-y) 35732791 PMC9217817

[11] Tsai SW, Chang WL, Pai FY, et al. Combination of enoxaparin and low-dose aspirin for thromboprophylaxis in selective patients after primary total joint arthroplasty in a Taiwanese population. J Chin Med Assoc 2023 86 923–929. (10.1097/jcma.0000000000000978) 37563769 PMC12718834

[12] Ritter M, Mamlin LA, Melfi CA, et al. Outcome implications for the timing of bilateral total knee arthroplasties. Clin Orthop Relat Res 1997 345 99–105. (10.1097/00003086-199712000-00014) 9418626

[13] Forster MC, Bauze AJ, Bailie AG, et al. A retrospective comparative study of bilateral total knee replacement staged at a one-week interval. J Bone Joint Surg Br 2006 88B 1006–1010. (10.1302/0301-620x.88b8.17862) 16877597

[14] Courtney PM, Melnic CM, Alosh H, et al. Is bilateral total knee arthroplasty staged at a one-week interval safe? A matched case control study. J Arthroplasty 2014 29 1946–1949. (10.1016/j.arth.2014.05.004) 24953946

[15] Chen AF, Rasouli MR, Vegari DN, et al. Staged bilateral total knee arthroplasty: time of the second side. J Knee Surg 2014 28 311–314. (10.1055/s-0034-1384215) 25068844

[16] Koh IJ, Kim GH, Kong CG, et al. The patient’s age and American society of anesthesiologists status are reasonable criteria for deciding whether to perform same-day bilateral TKA. J Arthroplasty 2015 30 770–775. (10.1016/j.arth.2014.12.004) 25512032

[17] Liu J, Elkassabany N, Poultsides L, et al. Staging bilateral total knee arthroplasty during the same hospitalization: the impact of timing. J Arthroplasty 2015 30 1172–1176. (10.1016/j.arth.2015.02.006) 25724110

[18] Chen J, Lee W, Chong H, et al. Identifying an ideal time frame for staged bilateral total knee arthroplasty to maximize functional outcome. J Knee Surg 2017 30 682–686. (10.1055/s-0036-1597273) 27898989

[19] Richardson SS, Kahlenberg CA, Blevins JL, et al. Complications associated with staged versus simultaneous bilateral total knee arthroplasty: an analysis of 7747 patients. Knee 2019 26 1096–1101. (10.1016/j.knee.2019.06.008) 31262633

[20] Crawford DA, Adams JB, Hurst JM, et al. Interval between staged bilateral total knee arthroplasties does not affect early medical or surgical complications. J Arthroplasty 2021 36 537–541. (10.1016/j.arth.2020.07.083) 32839059

[21] Mardani-Kivi M, Leili EK, Torfeh N, et al. Bilateral total knee arthroplasty: simultaneous versus staging in the same or in twice hospitalization. J Clin Orthop Trauma 2021 14 59–64. (10.1016/j.jcot.2020.09.023) 33717897 PMC7920008

[22] Xu H, Fei Z, Shang G, et al. A prospective comparative study of staged total knee arthroplasty: ninety-day versus seven-day interval. Int Orthop 2021 45 2885–2891. (10.1007/s00264-021-05037-x) 33825912

[23] Agarwal AR, Gu A, Wang KY, et al. Interval time of at least 6 weeks between bilateral total knee arthroplasties is associated with decreased postoperative complications. J Arthroplasty 2023 38 1063–1069. (10.1016/j.arth.2022.12.037) 36566996

[24] Sun K, Pi J, Wu Y, et al. The optimal period of staged bilateral total knee arthroplasty procedures under enhanced recovery: a retrospective study. Orthop Surg 2023 15 1249–1255. (10.1111/os.13684) 36794464 PMC10157719

[25] Chua HS, Whitehouse SL, Lorimer M, et al. Mortality and implant survival with simultaneous and staged bilateral total knee arthroplasty experience from the Australian orthopaedic association national joint replacement registry. J Arthroplasty 2018 33 3167–3173. (10.1016/j.arth.2018.05.019) 29908796

[26] Eldridge S, Campbell M, Campbell M, et al. 2016. Revised Cochrane Risk of Bias Tool for Randomized Trials (RoB 2.0): Additional Considerations for Cluster-Randomized Trials. Adelaide: University of South Australia. (https://www.unisa.edu.au/contentassets/72bf75606a2b4abcaf7f17404af374ad/rob2-0_cluster_parallel_guidance.pdf)

[27] Wells GA, Shea B, O’Connell D, et al. 2021. The Newcastle-Ottawa Scale (NOS) for assessing the quality of nonrandomised studies in meta-analyses. Ottawa, ON, Canada: Ottawa Health Research Institute. (www.ohri.ca/programs/clinical_epidemiology/oxford.asp)

[28] Hussain N, Chien T, Hussain F, et al. Simultaneous versus staged bilateral total knee arthroplasty: a meta-analysis evaluating mortality, peri-operative complications and infection rates. HSS J 2013 9 50–59. (10.1007/s11420-012-9315-7) 24426845 PMC3640720

[29] Roberts SSH, Teo WP & Warmington SA. Effects of training and competition on the sleep of elite athletes: a systematic review and meta-analysis. Br J Sports Med 2019 53 513–522. (10.1136/bjsports-2018-099322) 30217831

[30] Nikolakopoulou A, Higgins JPT, Papakonstantinou T, et al. CINeMA: an approach for assessing confidence in the results of a network meta-analysis. PLoS Med 2020 17 e1003082. (10.1371/journal.pmed.1003082) 32243458 PMC7122720

[31] Rücker G & Schwarzer G. Ranking treatments in frequentist network meta-analysis works without resampling methods. BMC Med Res Methodol 2015 15 58. (10.1186/s12874-015-0060-8) 26227148 PMC4521472

[32] Memtsoudis SG, Hargett M, Russell LA, et al. Consensus statement from the consensus conference on bilateral total knee arthroplasty group. Clin Orthop Relat Res 2013 471 2649–2657. (10.1007/s11999-013-2976-9) 23564364 PMC3705037

[33] Alshaikh AM, Alshaeri NM, Jamal R, et al. Mortality following simultaneous versus staged bilateral total knee arthroplasty: a systematic review and meta-analysis. Cureus 2023 15 e50823. (10.7759/cureus.50823) 38125692 PMC10732000

[34] Fu D, Li G, Chen K, et al. Comparison of clinical outcome between simultaneous-bilateral and staged-bilateral total knee arthroplasty: a systematic review of retrospective studies. J Arthroplasty 2013 28 1141–1147. (10.1016/j.arth.2012.09.023) 23518424

