# Supplementary material for: Optimal timing for bilateral total knee arthroplasty: comparing simultaneous and staged procedures at various intervals: a systematic review and network meta-analysis
Source: EFORT Open Rev. 2025 Jan 3;10(1):28–36. doi: 10.1530/EOR-2024-0070 (PMC11728876; doi:10.1530/EOR-2024-0070)
Supplement: Supplementary file 1 [file supplementary_materials.pdf]

Figure S1a-e. Network plots of secondary outcomes

Figure S1a. Neurologic complications

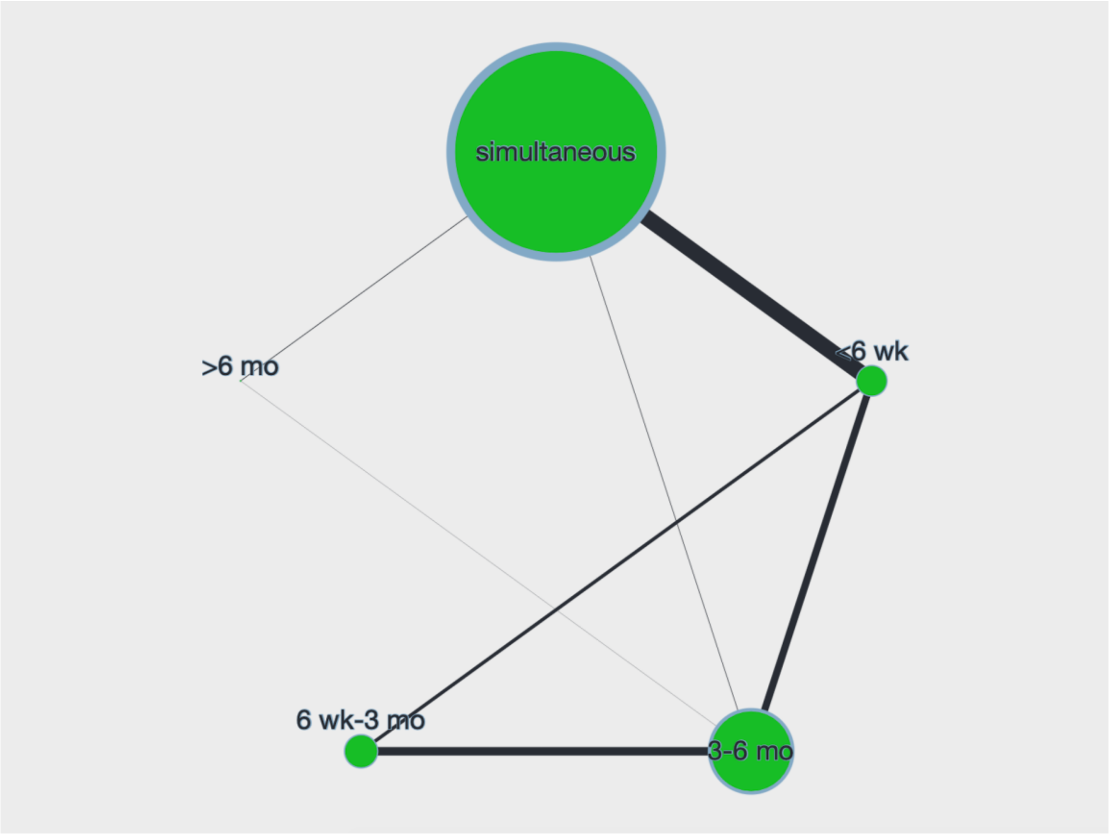

Figure S1b. Cardiovascular complications

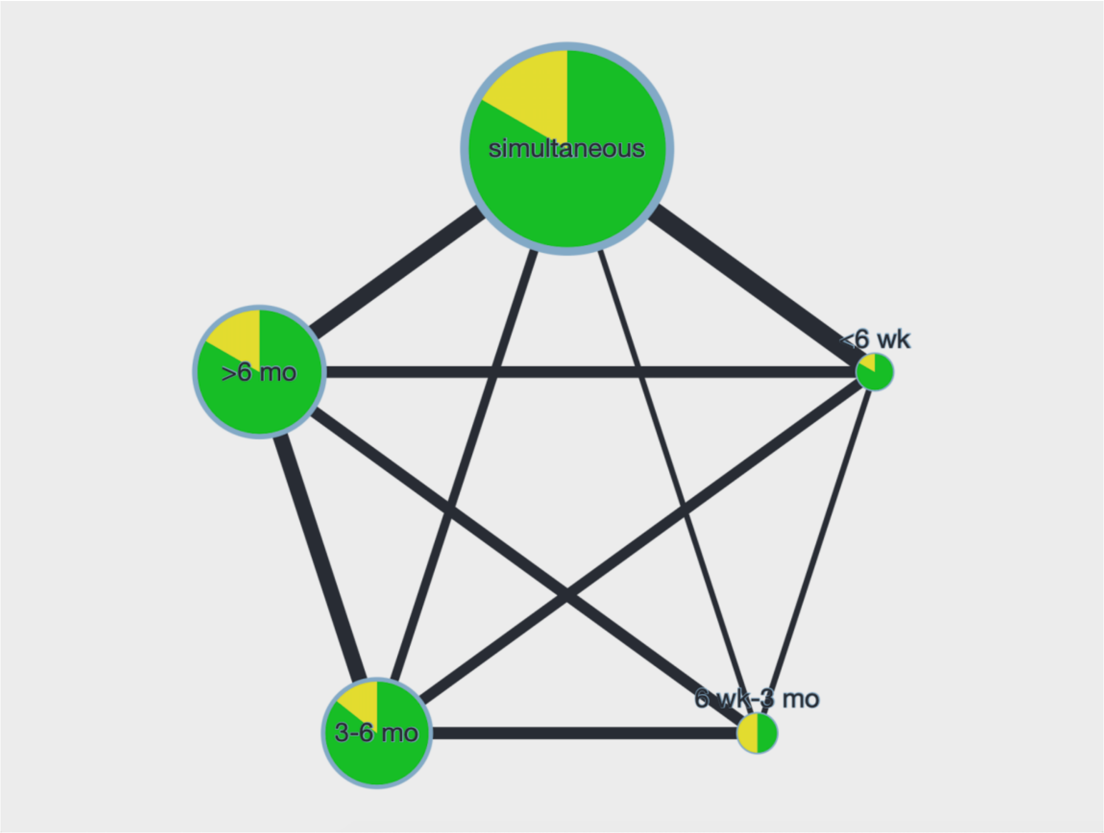

Figure S1c. Pulmonary complications

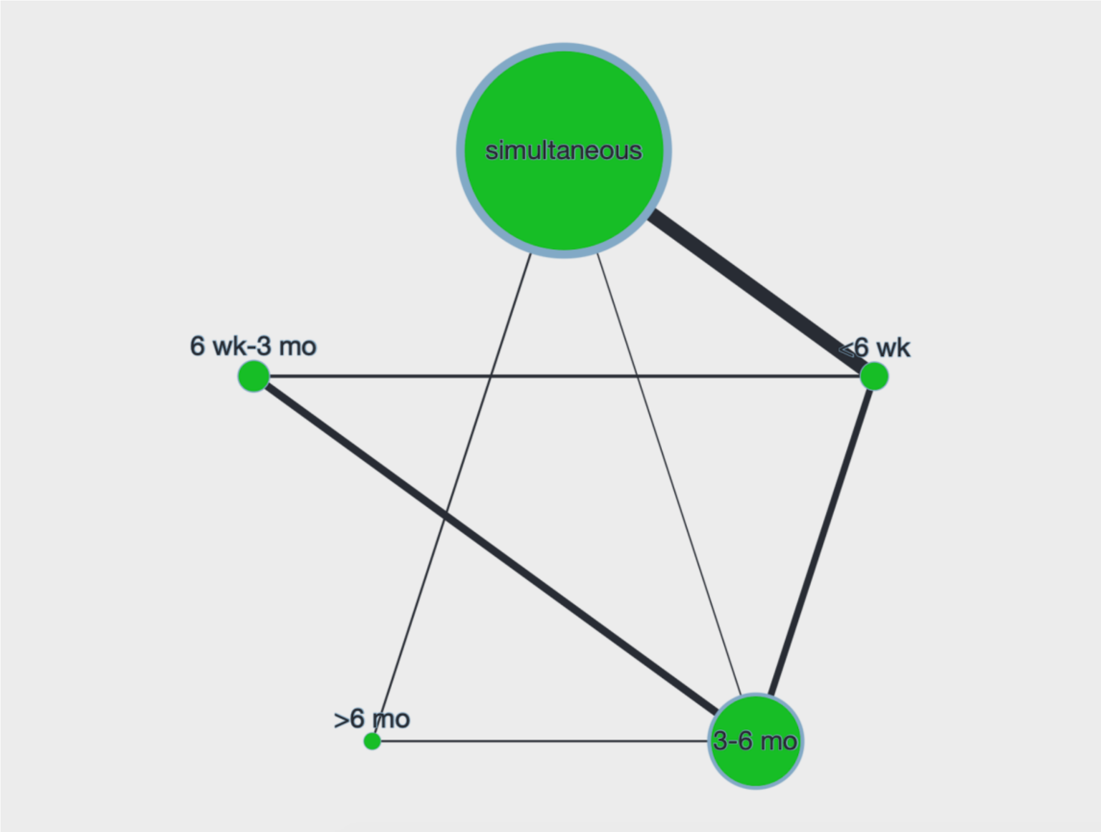

Figure S1d. Infection

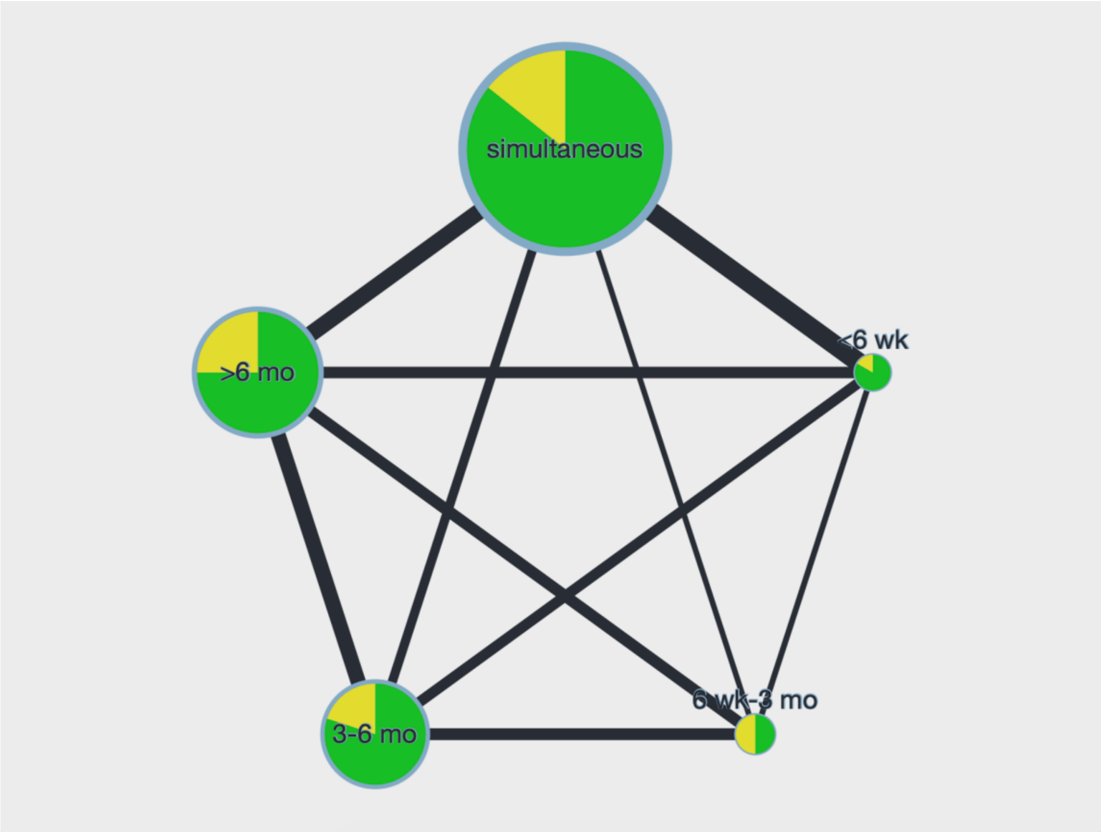

Figure S1e. Venous thromboembolism

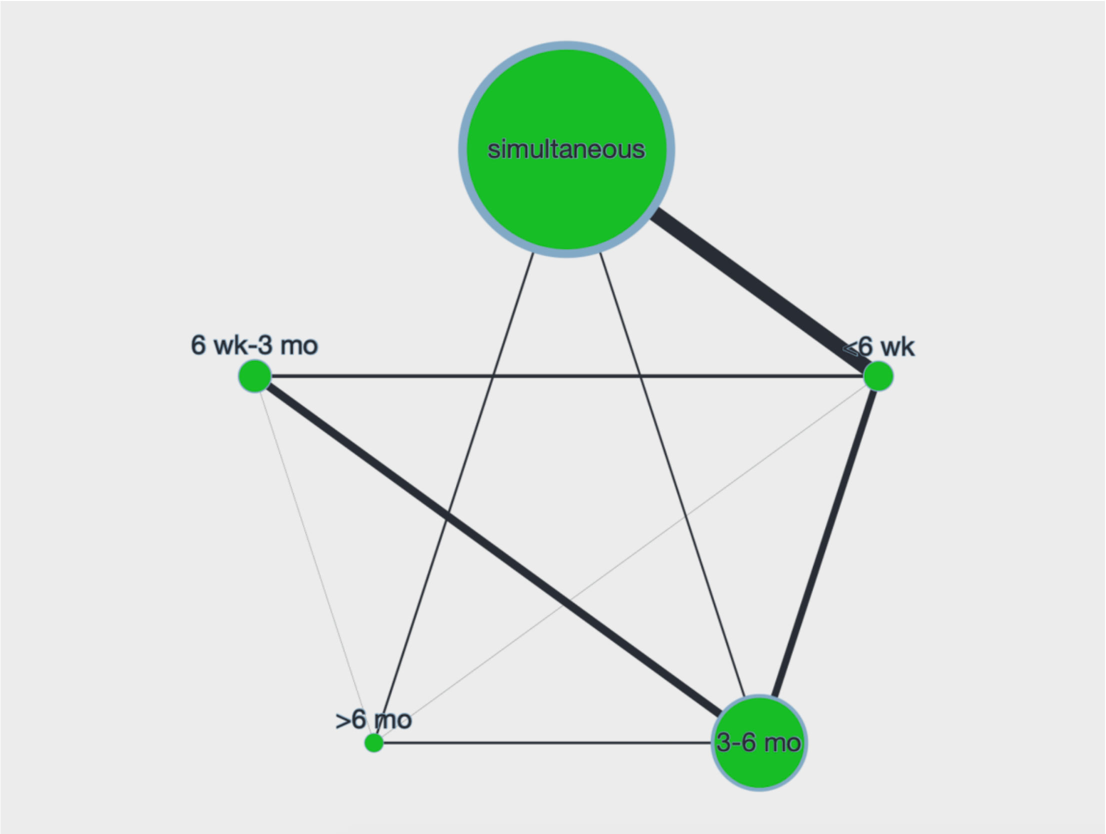

The size of the nodes represents the sample size in each group, while the thickness of the lines represents the sample size involved in the comparison between the two groups. The colors within the nodes indicate the assessed risk of bias for each group (green: low; yellow: moderate; red: high).

Figure S2a-e. Forest plots of secondary outcomes

Figure S2a. Neurologic complications

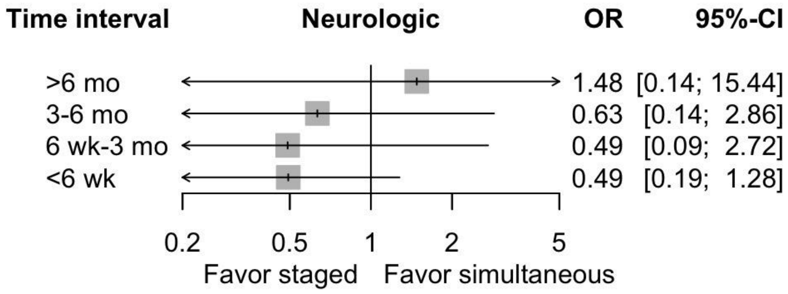

Figure S2b. Cardiovascular complications

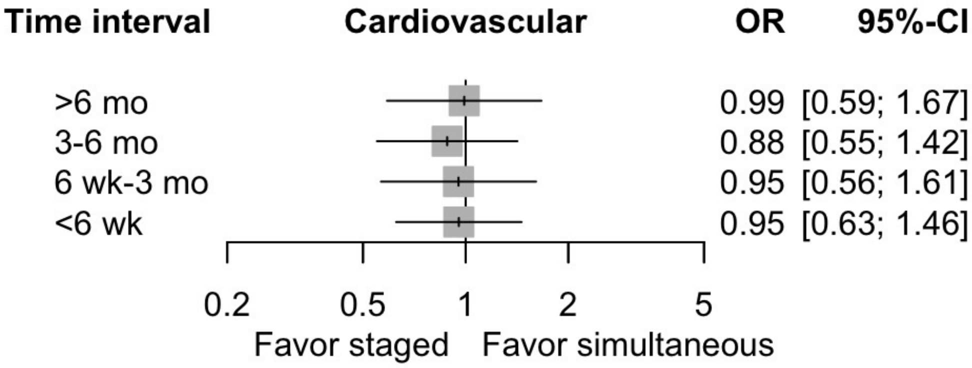

Figure S2c. Pulmonary complications

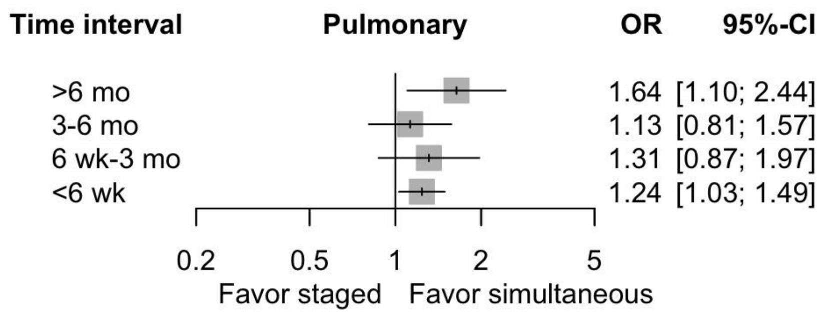

Figure S2d. Infection

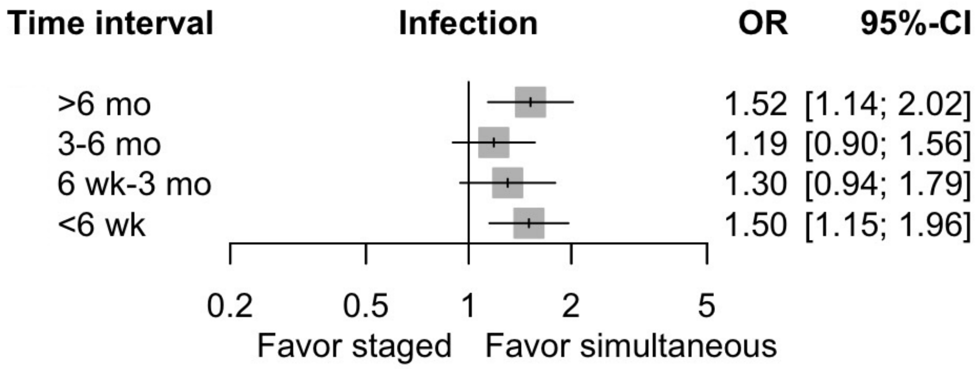

Figure S2e. Venous thromboembolism

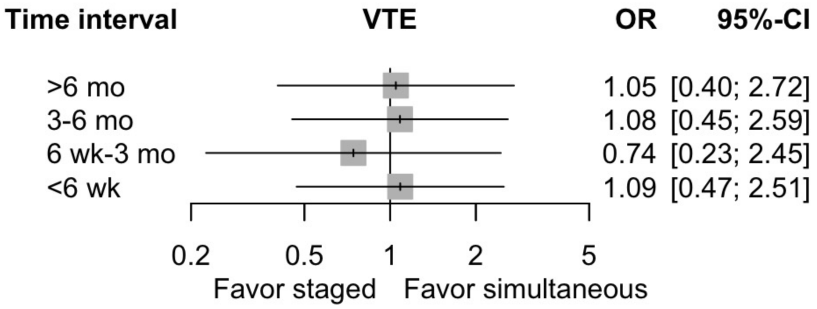

Each time interval was compared to the simultaneous interval, which served as the reference category.

OR = odds ratio; CI = confidence interval

Figure S3. Egger's test for primary outcomes

Figure S3a. Mortality

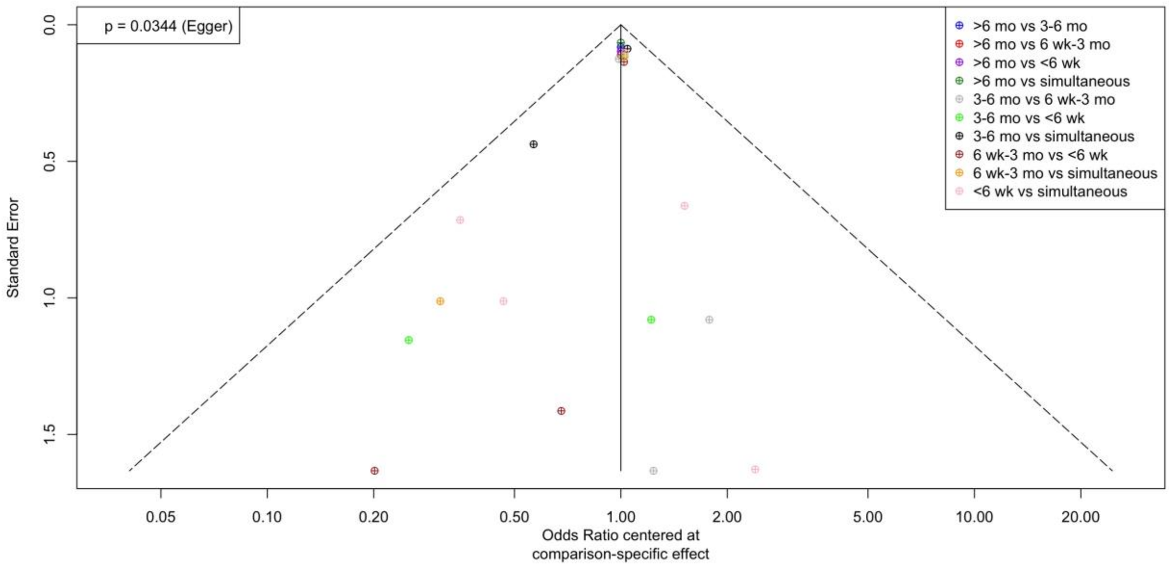

Figure S3b. Overall complications

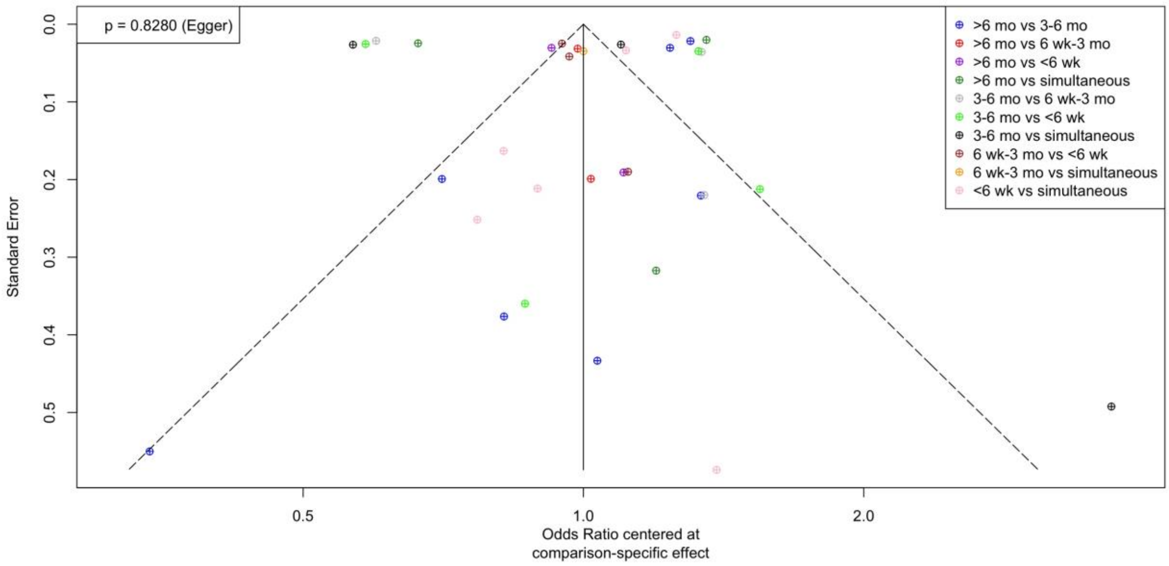

Table S1 PRISMA extension statement for reporting of systematic reviews incorporating network meta-analyses of health care interventions

**Section/Topic**	**Item #**	**Checklist Item**	**Reported on Page #**
**TITLE**			
Title	1	Identify the report as a systematic review *incorporating a network meta-analysis (or related form of meta-analysis).*	p1
**ABSTRACT**			
Structured summary	2	Provide a structured summary including, as applicable:<br>**Background:** main objectives<br>**Methods:** data sources; study eligibility criteria, participants, and interventions; study appraisal; and *synthesis methods, such as network meta-analysis.*<br>**Results:** number of studies and participants identified; summary estimates with corresponding confidence/credible intervals; *treatment rankings may also be discussed. Authors may choose to summarize pairwise comparisons against a chosen treatment included in their analyses for brevity.*<br>**Discussion/Conclusions:** limitations; conclusions and implications of findings.<br>**Other:** primary source of funding; systematic review registration number with registry name.	p2-3
**INTRODUCTION**			
Rationale	3	Describe the rationale for the review in the context of what is already known*, including mention of why a network meta-analysis has been conducted.*	p4
Objectives	4	Provide an explicit statement of questions being addressed, with reference to participants, interventions, comparisons, outcomes, and study design (PICOS)	p4
**METHODS**			
Protocol and registration	5	Indicate whether a review protocol exists and if and where it can be accessed (e.g., Web address); and, if available, provide registration information, including registration number.	p5
Eligibility criteria	6	Specify study characteristics (e.g., PICOS, length of follow-up) and report characteristics (e.g., years considered,	p5

		language, publication status) used as criteria for eligibility, giving rationale. *Clearly describe eligible treatments included in the treatment network, and note whether any have been clustered or merged into the same node (with justification).*	
Information sources	7	Describe all information sources (e.g., databases with dates of coverage, contact with study authors to identify additional studies) in the search and date last searched.	p5
Search	8	Present full electronic search strategy for at least one database, including any limits used, such that it could be repeated.	Supplementary Table S3
Study selection	9	State the process for selecting studies (i.e., screening, eligibility, included in systematic review, and, if applicable, included in the meta-analysis).	p5-6
Data collection process	10	Describe method of data extraction from reports (e.g., piloted forms, independently, in duplicate) and any processes for obtaining and confirming data from investigators.	p6
Data items	11	List and define all variables for which data were sought (e.g., PICOS, funding sources) and any assumptions and simplifications made.	p6
Geometry of the network	***S1***	Describe methods used to explore the geometry of the treatment network under study and potential biases related to it. This should include how the evidence base has been graphically summarized for presentation, and what characteristics were compiled and used to describe the evidence base to readers.	p6-7
Risk of bias within individual studies	12	Describe methods used for assessing risk of bias of individual studies (including specification of whether this was done at the study or outcome level), and how this information is to be used in any data synthesis.	p6-7
Summary measures	13	State the principal summary measures (e.g., risk ratio, difference in means). *Also describe the use of additional summary measures assessed, such as treatment rankings and surface under the cumulative ranking curve (SUCRA) values, as well as modified approaches used to present summary findings from meta-analyses.*	p7
Planned methods of	14	Describe the methods of handling data and combining results of studies for each network meta-analysis. This	p7

analysis		should include, but not be limited to:<br>• *Handling of multi-arm trials;*<br>• *Selection of variance structure;*<br>• *Selection of prior distributions in Bayesian analyses; and*<br>• *Assessment of model fit.*	
Assessment of Inconsistency	***S2***	Describe the statistical methods used to evaluate the agreement of direct and indirect evidence in the treatment network(s) studied. Describe efforts taken to address its presence when found.	p7
Risk of bias across studies	15	Specify any assessment of risk of bias that may affect the cumulative evidence (e.g., publication bias, selective reporting within studies).	p7
Additional analyses	16	Describe methods of additional analyses if done, indicating which were pre-specified. This may include, but not be limited to, the following:<br>• Sensitivity or subgroup analyses;<br>• Meta-regression analyses;<br>• *Alternative formulations of the treatment network; and*<br>• *Use of alternative prior distributions for Bayesian analyses (if applicable).*	p7
**RESULTS†**			
Study selection	17	Give numbers of studies screened, assessed for eligibility, and included in the review, with reasons for exclusions at each stage, ideally with a flow diagram.	p7,<br>Figure 1
Presentation of network structure	***S3***	Provide a network graph of the included studies to enable visualization of the geometry of the treatment network.	p7-8<br>Figure 2<br>Supplementary Figure S1
Summary of network geometry	***S4***	Provide a brief overview of characteristics of the treatment network. This may include commentary on the abundance of trials and randomised patients for the different interventions and pairwise comparisons in the network, gaps of evidence in the treatment network, and potential biases reflected by the network structure.	p7-8
Study	18	For each study, present characteristics for which data were	Table 1 and

characteristics		extracted (e.g., study size, PICOS, follow-up period) and provide the citations.	Supplementary Table S4
Risk of bias within studies	19	Present data on risk of bias of each study and, if available, any outcome level assessment.	Supplementary Table S5
Results of individual studies	20	For all outcomes considered (benefits or harms), present, for each study: 1) simple summary data for each intervention group, and 2) effect estimates and confidence intervals. *Modified approaches may be needed to deal with information from larger networks.*	p8-9
Synthesis of results	21	Present results of each meta-analysis done, including confidence/credible intervals. *In larger networks, authors may focus on comparisons versus a particular comparator (e.g. placebo or standard care), with full findings presented in an appendix. League tables and forest plots may be considered to summarize pairwise comparisons.* If additional summary measures were explored (such as treatment rankings), these should also be presented.	p8-9, Figure 3, Figure S2, Supplementary Tables S6
Exploration for inconsistency	***S5***	Describe results from investigations of inconsistency. This may include such information as measures of model fit to compare consistency and inconsistency models, *P* values from statistical tests, or summary of inconsistency estimates from different parts of the treatment network.	p9 Supplementary Table S7
Risk of bias across studies	22	Present results of any assessment of risk of bias across studies for the evidence base being studied.	p9 Supplementary Table S8
Results of additional analyses	23	Give results of additional analyses, if done (e.g., sensitivity or subgroup analyses, meta-regression analyses*, alternative network geometries studied, alternative choice of prior distributions for Bayesian analyses,* and so forth).	p9-10
**DISCUSSION**			
Summary of evidence	24	Summarize the main findings, including the strength of evidence for each main outcome; consider their relevance to key groups (e.g., healthcare providers, users, and policy-makers).	p10-12
Limitations	25	Discuss limitations at study and outcome level (e.g., risk of bias), and at review level (e.g., incomplete retrieval of identified research, reporting bias). *Comment on the validity of the assumptions, such as transitivity and consistency.*	p12-13

		Comment on any concerns regarding network geometry (e.g., avoidance of certain comparisons).	
Conclusions	26	Provide a general interpretation of the results in the context of other evidence, and implications for future research.	p13
**FUNDING**			
Funding	27	Describe sources of funding for the systematic review and other support (e.g., supply of data); role of funders for the systematic review. This should also include information regarding whether funding has been received from manufacturers of treatments in the network and/or whether some of the authors are content experts with professional conflicts of interest that could affect use of treatments in the network.	Title page p13

PICOS = population, intervention, comparators, outcomes, study design.

* Text in italics indicates wording specific to reporting of network meta-analyses that has been added to guidance from the PRISMA statement.

† Authors may wish to plan for use of appendices to present all relevant information in full detail for items in this section.

Table S2 PICOS for study selection

Question	The appropriate time interval for staged bilateral total knee arthroplasty
Population	Patient who was indicated for bilateral total knee arthroplasty
Intervention	Different time interval of staged bilateral total knee arthroplasty
Comparison	Simultaneous bilateral total knee arthroplasty
Outcome	Mortality, post-operative complications, neurologic complications, cardiovascular complications, pulmonary complications, infection, venous thromboembolism
Study design	Therapeutic

Table S3 Searching strategy in different databases

Table S3a Medline

Database	Medline (Ovid)	
Date	December 19, 2023	
Strategy		
#1	Bilateral total knee arthroplasty	2624
#2	Bilateral TKA	1203
#3	Bilateral total knee replacement	2182
#4	Bilateral TKR	145
#5	mesh.Exact("Arthroplasty, Replacement, Knee") AND bilateral	1646
#6	[S1] OR [S2] OR [S3] OR [S4] OR [S5]	2925
#7	simultaneous OR one-staged OR staggered OR staged OR staging OR non-simultaneous OR two-staged	588255
#8	[S6] AND [S7]	1038

Table S3b Embase

Database	Embase	
Date	December 19, 2023	
Strategy		
#1	'bilateral total knee' OR (bilateral AND ('total'/exp OR total) AND ('knee'/exp OR knee) AND arthroplast*)	2471
#2	bilateral AND tka	1066
#3	bilateral AND total AND knee AND replacement	1192
#4	bilateral AND tkr	198
#5	('total knee arthroplasty'/exp OR 'total knee arthroplasty') AND bilateral	2109
#6	#1 OR #2 OR #3 OR #4 OR #5	2638
#7	simultaneous OR one-staged OR staggered OR staged OR staging OR non-simultaneous OR two-staged	918672
#8	#6 and #7	794

Table S3c Cochrane

Database	Cochrane	
Date	December 19, 2023	
Strategy		
#1	MeSH descriptor: [Arthroplasty, Replacement, Knee] explode all trees	3492
#2	#1 AND bilateral	265
#3	(bilateral total knee arthroplast*):ti,ab,kw (Word variations have been searched)	562
#4	(bilateral TKA):ti,ab,kw (Word variations have been searched)	292
#5	(bilateral total knee replacement):ti,ab,kw (Word variations have been searched)	433
#6	(bilateral TKR):ti,ab,kw (Word variations have been searched)	51
#7	#2 OR #3 OR #4 OR #5 OR #6	684
#8	simultaneous OR one-staged OR staggered OR staged OR staging OR non-simultaneous OR two-staged	37609
#9	#7 AND #8	312

Table S3d Web of Science

Database	Web of Science	
Date	December 19, 2023	
Strategy		
#1	ALL=(Bilateral total knee arthroplasty) OR ALL=(Bilateral TKA) OR ALL=(Bilateral total knee replacement) OR ALL=(Bilateral TKR)	1733
#2	(ALL=(Bilateral total knee arthroplasty) OR ALL=(Bilateral TKA) OR ALL=(Bilateral total knee replacement) OR ALL=(Bilateral TKR)) AND (ALL=(Simultaneous) OR ALL=(one-staged) OR ALL=(staggered) OR ALL=(Staged) OR ALL=(staging) OR ALL=(non-simultaneous) OR ALL=(two-staged))	749

Table S4 Other characteristics of included studies

Author	Follow-up time	Patient size	Mean age	BMI	Female %	HTN %	DM %	Dyslipidemia %	CHF %	CKD %	Prior MI or CAD, %	Prior stroke or TIA, %	ASA	CCI	LOS
Ritter et al. (1997) (1)	>2 years	63030	73.0	N/A	65.8	N/A	N/A	N/A	N/A	N/A	N/A	N/A	N/A	N/A	19.01
Forster et al. (2006) (2)	4.22 years	102	N/A	N/A	52.0	N/A	N/A	N/A	N/A	N/A	N/A	N/A	1.95	N/A	13.53
Courtney et al. (2014) (3)	55.6 months	234	63.0	34.2	70.4	72.33	21.92	33.97	N/A	4.93	16.44	N/A	2.46	0.97	5.03
Liu et al. (2015) (4)	N/A	41664	65.3	N/A	59.1	N/A	N/A	N/A	N/A	N/A	N/A	N/A	N/A	N/A	4.44
Koh et al. (2015) (5)	>90 days	700	69.0	27.1	93.7	64.00	24.14	N/A	N/A	N/A	0.43	N/A	N/A	≦3	N/A
Chen et al. (2015) (6)	N/A	542	65.8	34.0	63.5	N/A	N/A	N/A	N/A	N/A	N/A	N/A	N/A	≦3	N/A
Yeh et al. (2017) (7)	2 years	306	66.3	28.2	83.0	N/A	N/A	N/A	N/A	N/A	N/A	N/A	N/A	2.63	N/A
Chua et al. (2018) (8)	15 years	36087	N/A	N/A	47.3	N/A	N/A	N/A	N/A	N/A	N/A	N/A	2.20	N/A	N/A
Richardson et al. (2019) (9)	N/A	7747	N/A	N/A	61.7	69.40	23.65	N/A	2.87	0.57	N/A	N/A	N/A	N/A	N/A
Crawford et al. (2021) (10)	10.7 months	1007	65.6	35.2	62.3	N/A	N/A	N/A	N/A	N/A	N/A	N/A	N/A	3.28	N/A
Mardani-Kivi et al. (2021) (11)	2 years	663	N/A	N/A	67.6	N/A	N/A	N/A	N/A	N/A	N/A	N/A	N/A	N/A	5.12
Xu et al. (2021) (12)	1 year	426	68.4	27.5	90.1	N/A	N/A	N/A	N/A	N/A	N/A	N/A	N/A	N/A	15.84
Sun et al.	N/A	281	66.	26	84.3	48.	15.	N/	N/	N/	N/	N/	2.6	N/	3.0

(2023) (13)			0	.6		75	66	A	A	A	A	A	6	A	0
Agarwal et al. (2023) (14)	N/A	25527	N/A	N/A	60.0	N/A	N/A	N/A	N/A	N/A	N/A	N/A	N/A	1.50	N/A
Chou et al. (2023) (15)	N/A	2016	72.1	28.1	77.7	N/A	N/A	N/A	N/A	N/A	N/A	N/A	1.07	N/A	8.02

BMI = Body mass index; HTN = hypertension; DM = diabetes mellitus; CHF = chronic heart failure; CKD = chronic kidney disease; MI = myocardial infarction; CAD = coronary artery disease; TIA = transient ischemic attack; ASA = American Society of Anesthesiologists; CCI = Charlson comorbidity index; LOS = Length of stay; N/A = not applicable.

References:

1. Ritter M, Mamlin LA, Melfi CA, Katz BP, Freund DA, Arthur DS. Outcome implications for the timing of bilateral total knee arthroplasties. Clin Orthop Relat Res. 1997(345):99-105.
2. Forster MC, Bauze AJ, Bailie AG, Falworth MS, Oakeshott RD. A retrospective comparative study of bilateral total knee replacement staged at a one-week interval. J Bone Joint Surg Br. 2006;88(8):1006-10.
3. Courtney PM, Melnic CM, Alosh H, Shah RP, Nelson CL, Israelite CL. Is bilateral total knee arthroplasty staged at a one-week interval safe? A matched case control study. J Arthroplasty. 2014;29(10):1946-9.
4. Liu J, Elkassabany N, Poultsides L, Nelson CL, Memtsoudis SG. Staging Bilateral Total Knee Arthroplasty During the Same Hospitalization: The Impact of Timing. J Arthroplasty. 2015;30(7):1172-6.
5. Koh IJ, Kim GH, Kong CG, Park SW, Park TY, In Y. The Patient's Age and American Society of Anesthesiologists Status Are Reasonable Criteria for Deciding Whether to Perform Same-Day Bilateral TKA. J Arthroplasty. 2015;30(5):770-5.
6. Chen AF, Rasouli MR, Vegari DN, Huang RC, Maltenfort MG, Parvizi J. Staged Bilateral Total Knee Arthroplasty: Time of the Second Side. J Knee Surg. 2015;28(4):311-4.
7. Yeh JZY, Chen JY, Lee WC, Chong HC, Pang HN, Tay DKJ, Chia SL, Lo NN, Yeo SJ. Identifying an Ideal Time Frame for Staged Bilateral Total Knee Arthroplasty to Maximize Functional Outcome. J Knee Surg. 2017;30(7):682-6.
8. Chua HS, Whitehouse SL, Lorimer M, De Steiger R, Guo L, Crawford RW. Mortality and Implant Survival With Simultaneous and Staged Bilateral Total Knee Arthroplasty Experience From the Australian Orthopaedic Association National Joint Replacement Registry. J Arthroplasty. 2018;33(10):3167-73.
9. Richardson SS, Kahlenberg CA, Blevins JL, Goodman SM, Sculco TP, Figgie MP, Sculco PK. Complications associated with staged versus simultaneous bilateral total knee arthroplasty: An analysis of 7747 patients. Knee. 2019;26(5):1096-101.
10. Crawford DA, Adams JB, Hurst JM, Morris MJ, Berend KR, Lombardi AV, Jr. Interval Between Staged Bilateral Total Knee Arthroplasties Does Not Affect Early Medical or Surgical Complications. J Arthroplasty. 2021;36(2):537-41.

11. Mardani-Kivi M, Leili EK, Torfeh N, Azari Z. Bilateral total knee arthroplasty: Simultaneous versus staging in the same or in twice hospitalization. J Clin Orthop Trauma. 2021;14:59-64.
12. Xu H, Fei Z, Shang G, Wang Y, Xiang S. A prospective comparative study of staged total knee arthroplasty: ninety-day versus seven-day interval. Int Orthop. 2021;45(11):2885-91.
13. Sun K, Pi J, Wu Y, Zeng Y, Xu J, Wu L, Li M, Shen B. The Optimal Period of Staged Bilateral Total Knee Arthroplasty Procedures under Enhanced Recovery: A Retrospective Study. Orthop Surg. 2023;15(5):1249-55.
14. Agarwal AR, Gu A, Wang KY, Harris AB, Campbell JC, Thakkar SC, Golladay GJ. Interval Time of at Least 6 Weeks Between Bilateral Total Knee Arthroplasties is Associated With Decreased Postoperative Complications. J Arthroplasty. 2023;38(6):1063-9.
15. Chou TA, Ma HH, Tsai CW, Tsai SW, Chen CF, Chiu FY, Wu PK, Chen WM. The safety and cost-analysis of simultaneous versus staged bilateral total knee arthroplasty in a Taiwan population. J Chin Med Assoc. 2023;86(5):494-8.

Table S5 Newcastle Ottawa Scale appraisal results for assessing the quality of non-randomized studies

Study author (year)	Selection				Comparability		Outcome			Total	Quality
	Representativeness of the exposed cohort	Selection of the non-exposed cohort	Ascertainment of exposure	Outcome of interest not present at start of study	Main factor	additional factor	Assessment of outcome	Sufficient follow-up time	Adequacy of follow up		
Ritter et al. (1997) (1)	1	1	1	1	0	1	1	0	0	6/9	Moderate
Forster et al. (2006) (2)	1	1	1	1	1	0	1	1	1	8/9	High
Courtney et al. (2014) (3)	1	1	1	1	1	0	1	1	1	8/9	High
Liu et al. (2015) (4)	1	1	1	1	1	1	1	0	1	8/9	High

Koh et al. (2015) (5)	1	1	1	1	1	0	1	1	1	8/9	High
Chen et al. (2015) (6)	1	0	1	1	1	1	1	0	1	7/9	High
Yeh et al. (2017) (7)	1	0	1	1	1	1	1	0	1	7/9	High
Chua et al. (2018) (8)	1	1	1	1	1	0	1	1	1	8/9	High
Richardson et al. (2019) (9)	1	1	1	1	1	1	1	1	1	9/9	High
Crawford et al. (2021) (10)	1	0	1	1	1	1	1	1	1	8/9	High
Mardani-Kivi et al. (2021) (11)	1	1	1	1	1	0	1	1	1	8/9	High
Xu et al. (2021) (12)	1	0	1	1	0	1	1	1	1	7/9	High
Sun et al. (2023) (13)	1	0	1	1	1	0	1	1	1	7/9	High
Agarwal et al. (2023) (14)	1	0	1	1	1	1	1	1	1	8/9	High
Chou et al (2023) (15)	1	1	1	1	0	0	1	1	0	8/9	High

References:

1. Ritter M, Mamlin LA, Melfi CA, Katz BP, Freund DA, Arthur DS. Outcome implications for the timing of bilateral total knee arthroplasties. Clin Orthop Relat Res. 1997(345):99-105.

2. Forster MC, Bauze AJ, Bailie AG, Falworth MS, Oakeshott RD. A retrospective comparative study of bilateral total knee replacement staged at a one-week interval. J Bone Joint Surg Br. 2006;88(8):1006-10.

3. Courtney PM, Melnic CM, Alosh H, Shah RP, Nelson CL, Israelite CL. Is bilateral total knee arthroplasty staged at a one-week interval safe? A matched case control study. J Arthroplasty. 2014;29(10):1946-9.

4. Liu J, Elkassabany N, Poultsides L, Nelson CL, Memtsoudis SG. Staging Bilateral Total Knee Arthroplasty During the Same Hospitalization: The Impact of Timing. J Arthroplasty. 2015;30(7):1172-6.

5. Koh IJ, Kim GH, Kong CG, Park SW, Park TY, In Y. The Patient's Age and American Society of Anesthesiologists Status Are Reasonable Criteria for Deciding Whether to Perform Same-Day Bilateral TKA. J Arthroplasty. 2015;30(5):770-5.

6. Chen AF, Rasouli MR, Vegari DN, Huang RC, Maltenfort MG, Parvizi J. Staged Bilateral Total Knee Arthroplasty: Time of the Second Side. J Knee Surg. 2015;28(4):311-4.

7. Yeh JZY, Chen JY, Lee WC, Chong HC, Pang HN, Tay DKJ, Chia SL, Lo NN, Yeo SJ. Identifying an Ideal Time Frame for Staged Bilateral Total Knee Arthroplasty to Maximize Functional Outcome. J Knee Surg. 2017;30(7):682-6.

8. Chua HS, Whitehouse SL, Lorimer M, De Steiger R, Guo L, Crawford RW. Mortality and Implant Survival With Simultaneous and Staged Bilateral Total Knee Arthroplasty Experience From the Australian Orthopaedic Association National Joint Replacement Registry. J Arthroplasty. 2018;33(10):3167-73.

9. Richardson SS, Kahlenberg CA, Blevins JL, Goodman SM, Sculco TP, Figgie MP, Sculco PK. Complications associated with staged versus simultaneous bilateral total knee arthroplasty: An analysis of 7747 patients. Knee. 2019;26(5):1096-101.

10. Crawford DA, Adams JB, Hurst JM, Morris MJ, Berend KR, Lombardi AV, Jr. Interval Between Staged Bilateral Total Knee Arthroplasties Does Not Affect Early Medical or Surgical Complications. J Arthroplasty. 2021;36(2):537-41.

11. Mardani-Kivi M, Leili EK, Torfeh N, Azari Z. Bilateral total knee arthroplasty: Simultaneous versus staging in the same or in twice hospitalization. J Clin Orthop Trauma. 2021;14:59-64.

12. Xu H, Fei Z, Shang G, Wang Y, Xiang S. A prospective comparative study of staged total knee arthroplasty: ninety-day versus seven-day interval. Int Orthop. 2021;45(11):2885-91.

13. Sun K, Pi J, Wu Y, Zeng Y, Xu J, Wu L, Li M, Shen B. The Optimal Period of Staged Bilateral Total Knee Arthroplasty Procedures under Enhanced Recovery: A Retrospective Study. Orthop Surg. 2023;15(5):1249-55.

14. Agarwal AR, Gu A, Wang KY, Harris AB, Campbell JC, Thakkar SC, Golladay GJ. Interval Time of at Least 6 Weeks Between Bilateral Total Knee Arthroplasties is Associated With Decreased Postoperative Complications. J Arthroplasty. 2023;38(6):1063-9.

15. Chou TA, Ma HH, Tsai CW, Tsai SW, Chen CF, Chiu FY, Wu PK, Chen WM. The safety and cost-analysis of simultaneous versus staged bilateral total knee arthroplasty in a Taiwan population. J Chin Med Assoc. 2023;86(5):494-8.

Table S6 League table comparing outcomes between staged BTKA with different time intervals and simultaneous BTKA

Table S6a Neurologic complications

**>6 months**	0.31 (0.01; 9.90)	N/A	N/A	2.65 (0.23; 30.33)
2.34 (0.17; 31.45)	**3-6 months**	1.14 (0.27; 4.84)	0.82 (0.21; 3.24)	8.54 (0.31; 234.15)
3.01 (0.19; 48.35)	1.29 (0.32; 5.24)	**6 weeks - 3 months**	0.85 (0.18; 3.97)	N/A
3.00 (0.26; 35.14)	1.28 (0.36; 4.62)	0.99 (0.23; 4.35)	**<6 weeks**	0.39 (0.15; 1.05)
1.48 (0.14; 15.44)	0.63 (0.14; 2.86)	0.49 (0.09; 2.72)	0.49 (0.19; 1.28)	**Simultaneous**

Table S6b Cardiovascular complications

**>6 months**	0.96 (0.57; 1.62)	1.51 (0.75; 3.02)	1.65 (0.82; 3.32)	0.99 (0.56; 1.76)
1.12 (0.69; 1.84)	**3-6 months**	0.90 (0.55; 1.46)	0.94 (0.58; 1.52)	0.87 (0.49; 1.55)
1.04 (0.59; 1.82)	0.93 (0.58; 1.48)	**6 weeks - 3 months**	1.09 (0.67; 1.79)	0.79 (0.39; 1.59)
1.04 (0.61; 1.75)	0.92 (0.60; 1.43)	1.00 (0.62; 1.60)	**<6 weeks**	0.95 (0.60; 1.51)
0.99 (0.59; 1.67)	0.88 (0.55; 1.42)	0.95 (0.56; 1.61)	0.95 (0.63; 1.46)	**Simultaneous**

Table S6c Pulmonary complications

**>6 months**	1.40 (0.97; 2.01)	N/A	N/A	1.81 (1.13; 2.91)*
1.45 (1.03; 2.05)*	**3-6 months**	0.85 (0.64; 1.15)	0.84 (0.56; 1.25)	1.26 (0.76; 2.09)
1.25 (0.81; 1.94)	0.86 (0.64; 1.15)	**6 weeks - 3 months**	1.02 (0.66; 1.60)	N/A
1.32 (0.88; 1.98)	0.91 (0.66; 1.26)	1.06 (0.71; 1.57)	**<6 weeks**	1.22 (1.00; 1.47)*
1.64 (1.10; 2.44)*	1.13 (0.81; 1.57)	1.31 (0.87; 1.97)	1.24 (1.03; 1.49)*	**Simultaneous**

Table S6d Infection

**>6 months**	1.18 (0.88; 1.60)	1.21 (0.80; 1.85)	1.01 (0.67; 1.54)	1.53 (1.13; 2.07)*
1.28 (0.96; 1.70)	**3-6 months**	0.86 (0.63; 1.17)	0.75 (0.55; 1.03)	1.28 (0.94; 1.76)
1.17 (0.83; 1.63)	0.91 (0.68; 1.22)	**6 weeks - 3 months**	0.86 (0.62; 1.19)	1.00 (0.65; 1.52)
1.01 (0.74; 1.38)	0.79 (0.60; 1.04)	0.87 (0.64; 1.18)	**<6 weeks**	1.35 (1.00; 1.81)*
1.52 (1.14; 2.02)*	1.19 (0.90; 1.56)	1.30 (0.94; 1.79)	1.50 (1.15; 1.96)*	**Simultaneous**

Table S6e Venous thromboembolism

**>6 months**	0.82 (0.34; 1.96)	4.21 (0.55; 32.05)	1.79 (0.33; 9.67)	0.91 (0.28; 3.01)
0.97 (0.42; 2.22)	**3-6 months**	1.02 (0.34; 3.08)	0.60 (0.21; 1.72)	1.65 (0.54; 5.06)
1.41 (0.43; 4.63)	1.46 (0.52; 4.10)	**6 weeks - 3 months**	0.55 (0.19; 1.63)	N/A
0.97 (0.36; 2.58)	1.00 (0.43; 2.33)	0.68 (0.24; 1.94)	**<6 weeks**	0.80 (0.28; 2.29)
1.05 (0.40; 2.72)	1.08 (0.45; 2.59)	0.74 (0.23; 2.45)	1.09 (0.47; 2.51)	**Simultaneous**

The league table presents odds ratios (OR) with 95% CI for comparing different time intervals between bilateral knee arthroplasties. The upper half of the triangle shows odds ratios (OR) for direct comparisons available within our network; 'N/A' indicates no direct comparisons were available. The lower half of the triangle includes ORs for estimated effect sizes based on both direct and indirect comparisons. In the upper grids, each box compares the time interval defined by the row with that defined by the column. Conversely, in the lower grids, each box compares the time interval defined by the column with that defined by the row. Symbol * denotes statistically significant difference.

Table S7 Consistency and publication bias in the comparisons among the five arms regarding time intervals in primary outcomes

Outcomes	Consistency P-value	Egger's test P-value
Mortality	0.8564	0.0344*
Overall complications	0.3835	0.8280

Symbol * denotes statistically significant difference.

Table S8a CINeMA evaluation of confidence in the findings from network meta-analysis for mortality

Comparison	Number of studies	Within-study bias	Reporting bias	Indirectness	Imprecision	Heterogeneity	Incoherence	Confidence rating
<6 weeks vs. 6 weeks-3 months	3	Some concerns	Low risk	No concerns	No concerns	No concerns	No concerns	Moderate
<6 weeks vs. 3-6 months	3	Some concerns	Low risk	No concerns	No concerns	No concerns	No concerns	Moderate
<6 weeks vs. >6 months	1	Some concerns	Low risk	No concerns	No concerns	No concerns	No concerns	Moderate
<6 weeks	5	Some	Low	No	No	No	No	Moderat

vs. simultaneous		concerns	risk	concerns	concerns	concerns	concerns	e
6 weeks-3 months vs. 3-6 months	3	Some concerns	Low risk	No concerns	No concerns	No concerns	Some concerns	Low
6 weeks-3 months vs. >6 months	1	Some concerns	Low risk	No concerns	No concerns	No concerns	No concerns	Moderate
6 weeks-3 months vs. simultaneous	2	Some concerns	Low risk	No concerns	No concerns	No concerns	No concerns	Moderate
3-6 months vs. >6 months	1	Some concerns	Low risk	No concerns	No concerns	No concerns	No concerns	Moderate
3-6 months vs. simultaneous	2	Some concerns	Low risk	No concerns	No concerns	No concerns	No concerns	Moderate
>6 months vs. simultaneous	1	Some concerns	Low risk	No concerns	No concerns	No concerns	No concerns	Moderate

Table S8b CINeMA evaluation of confidence in the findings from network meta-analysis for overall complications

Comparison	Number of studies	Within-study bias	Reporting bias	Indirectness	Imprecision	Heterogeneity	Incoherence	Confidence rating
<6 weeks vs. 6 weeks-3 months	3	No concerns	Low risk	No concerns	No concerns	Some concerns	No concerns	Moderate
<6 weeks	4	No	Low	No	No	Some	No	Moderat

vs. 3-6 months		concerns	risk	concerns	concerns	concerns	concerns	e
<6 weeks vs. >6 months	2	No concerns	Low risk	No concerns	No concerns	Some concerns	Some concerns	Low
<6 weeks vs. simultaneous	6	No concerns	Low risk	No concerns	No concerns	No concerns	No concerns	High
6 weeks-3 months vs. 3-6 months	3	No concerns	Low risk	No concerns	No concerns	Some concerns	No concerns	Moderate
6 weeks-3 months vs. >6 months	2	No concerns	Low risk	No concerns	No concerns	Some concerns	Some concerns	Low
6 weeks-3 months vs. simultaneous	1	No concerns	Low risk	No concerns	No concerns	Some concerns	No concerns	Moderate
3-6 months vs. >6 months	7	No concerns	Low risk	No concerns	No concerns	No concerns	Some concerns	Moderate
3-6 months vs. simultaneous	3	No concerns	Low risk	No concerns	No concerns	Some concerns	No concerns	Moderate
>6 months vs. simultaneous	3	No concerns	Low risk	No concerns	No concerns	Some concerns	No concerns	Moderate

Table S9 Ranking with the P-score in different complications

Mortality		Complications		Neurologic		Cardiovascular	
Time interval	P-score	Time interval	P-score	Time interval	P-score	Time interval	P-score
3-6 months	0.7849	3-6 months	0.7077	<6 weeks	0.7205	3-6 months	0.6593
>6 months	0.7592	>6 months	0.6548	6 weeks-3 months	0.6789	6 weeks-3 months	0.5009
6 weeks-3 months	0.7004	6 weeks-3 months	0.4518	3-6 months	0.5441	<6 weeks	0.4992
<6 weeks	0.1463	simultaneous	0.3528	simultaneous	0.2962	>6 months	0.4325
simultaneous	0.1092	<6 weeks	0.3329	>6 months	0.2604	simultaneous	0.4081

Pulmonary		Infection		VTE	
Time interval	P-score	Time interval	P-score	Time interval	P-score
simultaneous	0.9110	simultaneous	0.9570	6 weeks-3 months	0.7312
3-6 months	0.6972	3-6 months	0.6886	simultaneous	0.4993
<6 weeks	0.4545	6 weeks-3 months	0.4888	>6 months	0.4519
6 weeks-3 months	0.3697	<6 weeks	0.1892	3-6 months	0.4095
>6 months	0.0677	>6 months	0.1764	<6 weeks	0.4081

The P-score is calculated using a frequentist method to rank treatments by measuring the certainty that one treatment is superior to another, averaged across all competing treatments. Representing a probability value, higher P-scores indicate a greater likelihood that a particular treatment is more effective compared to others in the dataset.
